# Psychosocial Interventions in the Rehabilitation and the Management of Psychosis and Schizophrenia: A Systematic Review on Digitally-Delivered Interventions

**DOI:** 10.62641/aep.v53i2.1851

**Published:** 2025-03-05

**Authors:** Laura Orsolini, Giulio Longo, Umberto Volpe

**Affiliations:** ^1^Unit of Clinical Psychiatry, Department of Clinical Neurosciences/DIMSC, Polytechnic University of Marche, 60126 Ancona, Italy

**Keywords:** digital psychiatry, psychoeducation, psychosocial interventions, schizophrenia, psychosis, rehabilitation, Web-based, video

## Abstract

**Background:**

Schizophrenia and psychotic disorders are disabling, complex and severe psychiatric conditions, which may pose a significant therapeutic challenge. Integrating current psychopharmacological treatment with psychosocial interventions demonstrated a higher efficacy in terms of prognosis. However, most schizophrenia or psychotic patients may have restricted or no access to evidence-based psychosocial interventions, mainly due to poor dissemination of specialized interventions or stigma. Therefore, we aim to systematically review all studies about the current evidence on the feasibility, acceptability, efficacy, effectiveness, and benefits of digitally-delivered psychoeducational and psychosocial interventions for individuals suffering from schizophrenia or psychotic disorders.

**Methods:**

A systematic literature review was conducted of the literature from 2000 to 2024 according to the Preferred Reporting Items for Systematic Reviews and Meta-Analyses (PRISMA) guidelines, by using PubMed-MEDLINE, Scopus and OVID databases and combining the search approach using both free text terms and Medical Subject Headings (MESH) headings for the topics “*psychoeducation*”, “*psychosocial intervention*” and “*psychosis*” and “*schizophrenia*”.

**Results:**

Out of a total of 3042 reviewed papers, 69 studies were included here. The interventions included web-based family and individual psychoeducation, integrated web-based therapy, social networking, peer and expert moderation, virtual reality-assisted and mobile-based psychosocial interventions. Results showed that digitally-delivered interventions have a positive effect in ensuring the continuity and maintenance of the effectiveness of psychosocial treatments, by providing personalized, flexible, and evidence-based interventions to patients with psychosis and/or schizophrenia. At the same time, the studies included demonstrated the acceptability and feasibility of this kind of intervention in clinical practice.

**Conclusions:**

Digital interventions have the potential to deliver non-stigmatizing, constantly available psychosocial and psychoeducational interventions in psychosis and schizophrenia by increasing access to mental health care and not costly interventions. However, further randomized controlled trials (RCTs) and observational studies should compare and evaluate the effectiveness and feasibility of web-based vs. face-to-face psychosocial interventions amongst schizophrenia and psychosis individuals.

## Introduction

Schizophrenia and schizophrenia-related disorders (i.e., schizoaffective disorders, schizophreniform disorder and other psychotic disorders) are severe mental illnesses (SMIs) affecting more than 24 million people globally, often resulting in long-term disabilities and diminished cognitive, social, and emotional functioning [1]. In particular, social cognition impairment may hugely hinder functional recovery in individuals affected by schizophrenia and other psychotic disorders, as they negatively impact interpersonal relationships, vocational functioning and community adjustment [2]. Beyond psychopharmacological treatments, psychosocial and psychoeducation interventions are currently seen as essential in improving symptom management, quality of life, prognosis, functional recovery, and relapse prevention, particularly amongst young patients with first episode psychosis (FEP) [3, 4, 5]. Psychosocial interventions currently consist of a wide range of techniques and approaches, including at least different forms of assertive outreach programs, cognitive behavioural therapy for psychosis (CBTp), medication adherence and family support, which in turn may comprise a combination of individual- and family-based psychoeducation interventions [6, 7, 8, 9, 10, 11]. However, despite the availability of many evidence-based psychosocial interventions for psychosis, most patients with schizophrenia or psychosis may have limited or no access to such interventions [12]. In fact, mainly due to costly delivery, geographic barriers and logistic limitations, poor dissemination of specialized interventions or stigma associated with mental health treatment, many schizophrenia and psychosis patients may display limited help seeking and treatment adherence [12].

The digital revolution assisted in better interaction and easy accessibility of online tools also amongst patients with psychosis and schizophrenia. These patients have effectively demonstrated the ability to build virtual social connections and relationships, which have been shown to help them overcome their troubles with social interaction and social cognition [13]. The rise of new technologies facilitated the use of different electronic applications, social networks and other online devices, including smartphones, which have been largely disseminated to deliver alternative and/or complementary tools to individuals with psychosis and schizophrenia [14]. Digitally-based psychosocial and psychoeducation interventions may help in ensuring the continuity and maintenance of the effectiveness of psychosocial treatments [3]. In fact, digital tools are potentially able to provide evidence-based interventions, personalized and flexible to patients with psychosis and/or schizophrenia, even in their own homes [3]. Available evidence demonstrated that up to 90% of people with FEP have access to a smartphone [13, 15, 16]. Moreover, smartphone-based interventions are considered flexible, attractive and safe among young patients with FEP or psychosis [3, 17, 18]. Despite several studies have demonstrated that digitally-delivered interventions are effective for treating several mental disorders [19, 20, 21, 22], few and contrasting studies are currently available about the application of Internet-, mobile-, and virtual reality-based treatments in psychotic disorders and schizophrenia [23, 24, 25, 26, 27].

Overall, there is a substantial gap between needed psychosocial rehabilitation services and their availability—the Coronavirus disease-2019 (COVID-19) pandemic, spread worldwide since December 2019, may be an opportunity. Many patients are not situated by skilled centres and clinicians yet have not been reached by video care. COVID-19 has led to unprecedented changes in delivering mental health care and facilitated the transition from in-person to digital diagnosis and therapy, particularly amongst youths [28, 29, 30, 31, 32]. One could argue that the current COVID-19 pandemic may have increased the need for services, even in urban settings, and accelerated the spread of psychosocial interventions delivered online. Indeed, the COVID-19 pandemic stimulated the growth of further studies specifically designed to address the digital necessities of rehabilitation and functional recovery of individuals affected with schizophrenia and psychosis, as well as the psychoeducational needs of their parents and caregivers. Nevertheless, despite some studies [23, 24, 25, 26, 27] demonstrated that online psychosocial and psychoeducation treatments are overly feasible and safe, particularly amongst young people with FEP or psychosis or schizophrenia, a more meticulous examination of the emerging evidence on the potential of digitally-based tools in delivering psychosocial interventions to support psychosis and schizophrenia treatment and recovery is needed.

The present study provides an overview of digitally-delivered mental health interventions and aims to systematically review studies with current evidence on the feasibility, acceptability, efficacy, effectiveness and benefits of digitally-delivered psychoeducational and psychosocial interventions for individuals suffering from schizophrenia or psychotic disorders.

## Materials and Methods

###  Search Sources and Strategies

**Fig. 1.**
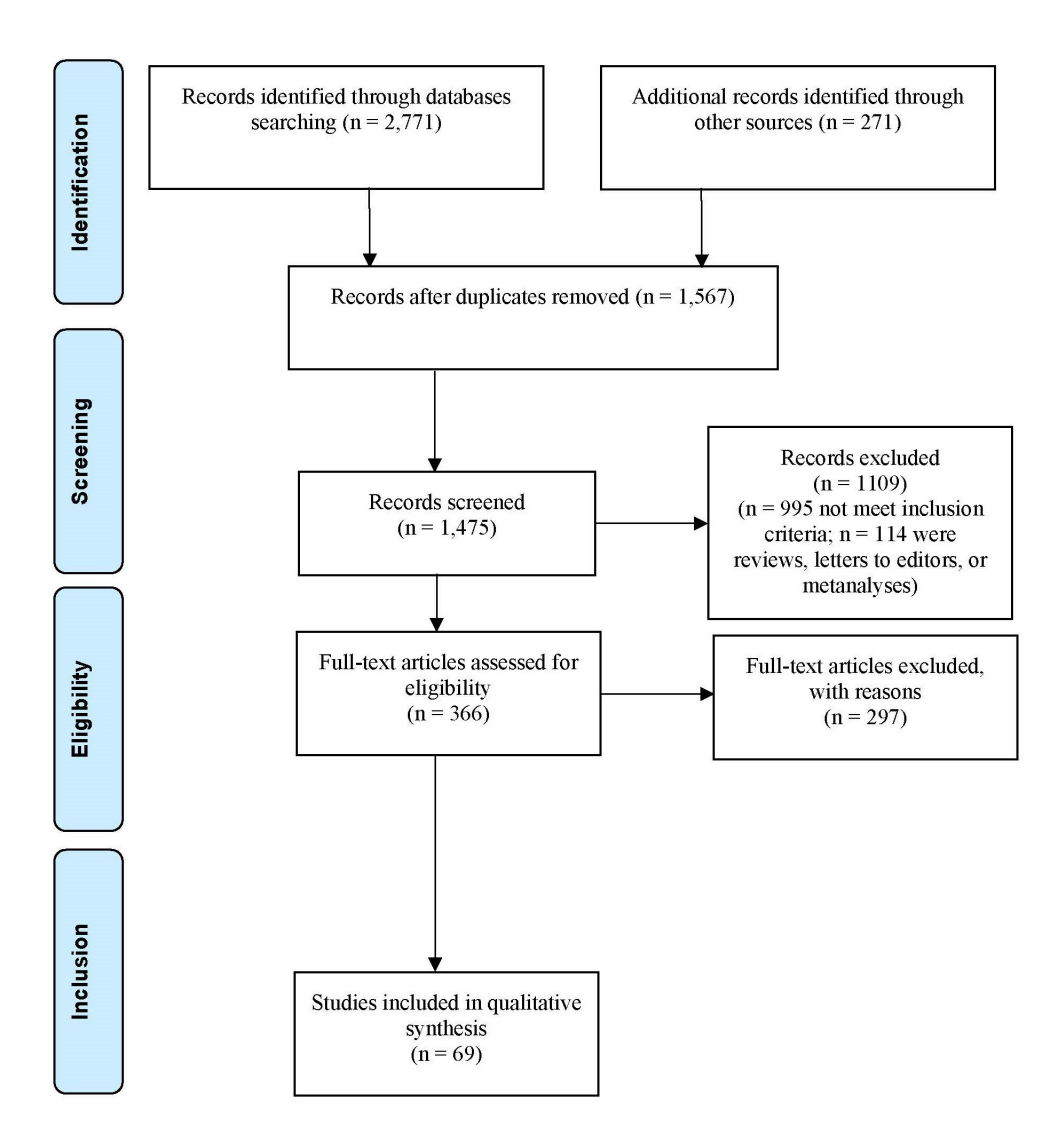
**Preferred Reporting Items for Systematic Reviews and Meta-Analyses (PRISMA) flow diagram**.

We conducted a systematic review according to the Preferred Reporting Items for Systematic Reviews and Meta-Analyses (PRISMA) guidelines (**Supplementary file 1**) [33]. Literature searches were conducted using the PubMed-MEDLINE, OVID and Scopus databases. The search strategy involved a combination of free text terms and expanded Medical Subject Headings (MESH) headings related to the topics of *Psychoeducation* and *Psychosocial Interventions* delivered online in Psychosis and Schizophrenia as follows: ((*psychoeducation* [Title/Abstract]) OR (*psychosocial* [Title/Abstract])) AND ((*web* [Title/Abstract]) OR (*digital* [Title/Abstract]) OR (*online* [Title/Abstract])) AND ((*psychosis* [Title/Abstract]) OR (*schizophrenia* [Title/Abstract])) (Fig. 1). An initial screening was conducted using titles and abstracts, followed by a second screening that involved reviewing the full texts. All studies published from 2000 through 12 September 2024, were included here without time limitation. Additionally, secondary searches were conducted by reviewing the reference lists of all eligible and pertinent articles, consulting with field experts, and performing manual searches.

###  Study Selection

We considered studies evaluating all online psychoeducational and/or psychosocial interventions delivered on subjects affected with psychosis and/or Schizophrenia-related disorders or their family members/family. Working independently and in pairs, LO and GL read the papers and determined whether they met the inclusion criteria. Duplicate publications were properly excluded. Studies needed to satisfy the following criteria to be considered for inclusion in this review: (a) empirical and peer-reviewed study; (b) at least an abstract with estimates and/or full results published in English, even though the paper is written in a not-English language; (c) investigate the efficacy and/or effectiveness of online/web-based/digital psychoeducational and/or psychosocial interventions in schizophrenia and/or psychotic disorders; (d) human studies. All articles identified by the data sources, reporting original data related to online psychoeducation and/or psychosocial interventions in schizophrenia or psychotic disorders, were evaluated in the present review. All experimental and observational study designs were considered for inclusion, except for case reports. In the present systematic review, randomized controlled clinical trials were prioritized. Narrative and systematic reviews, book chapters, and letters to the editor were excluded from the analysis, even though they were considered for looking for further relevant references to be included.

###  Data Extraction and Management

LO and GL extracted data pertaining to participant demographics, intervention specifics, and study outcomes. Discrepancies in data extraction were addressed through collaborative discussion and consensus with a third, independent researcher (UV). A custom-designed spreadsheet was utilized for data collection.

## Results

An initial search using the specified keywords produced a total of 3042 results (Table 1). After removing duplicates (n = 1567), a further 995 papers were excluded as they did not meet the inclusion criteria listed above, while 114 papers were excluded because they were reviews, letters to editors, or metanalyses. Amongst 366 remaining studies screened for eligibility depending on their abstract, 297 were not included because they were not pertinent to the topic of the present investigation. Finally, a total of 69 papers were included and accounted for in our analysis. Table 2 (Ref. [3, 6, 7, 34, 35, 36, 37, 38, 39, 40, 41, 42, 43, 44, 45, 46, 47, 48, 49, 50, 51, 52, 53, 54, 55, 56, 57, 58, 59, 60, 61, 62, 63, 64, 65, 66, 67, 68, 69, 70, 71, 72, 73, 74, 75, 76, 77, 78, 79, 80, 81, 82, 83, 84, 85, 86, 87, 88, 89, 90, 91, 92, 93, 94, 95, 96, 97, 98, 99]) summarizes all main characteristics (main outcomes, study design, findings and sample size) of all studies retrieved here, including those studies not strictly targeted to schizophrenia and psychosis but dealing with schizophrenia-related topics [34, 35, 36]. Studies on digital psychosocial interventions can be classified according to several characteristics: (a) type of intervention (i.e., program-based, smartphone-based, virtual-reality-assisted, etc.); (b) target population (i.e., patients, caregivers or general population); (c) type of diagnosis (i.e., psychosis spectrum disorders or schizophrenia). For ease, all included studies were reported in Table 2 and classified according to the above-mentioned characteristics. Table 3 (Ref. [3, 6, 7, 34, 35, 36, 37, 38, 39, 40, 41, 42, 43, 44, 45, 46, 47, 48, 49, 50, 51, 52, 53, 54, 55, 56, 57, 58, 59, 60, 61, 62, 63, 64, 65, 66, 67, 68, 69, 70, 71, 72, 73, 74, 75, 76, 77, 78, 79, 80, 81, 82, 83, 84, 85, 86, 87, 88, 89, 90, 91, 92, 93, 94, 95, 96, 97, 98, 99]) summarises the specifications of the different digital interventions included in this review.

**Table 1.** **MEDLINE search strategy**.

SET	MEDLINE
1	Psychoeducation
2	Psychosocial Intervention
3	Sets 1–2 were combined with “OR”
4	Digital
5	Web
6	Online
7	Sets 4–6 were combined with “OR”
8	*Psychosis*
9	*Schizophrenia*
10	Sets 8–9 were combined with “OR”
11	Sets 3, 7 and 10 were combined with “AND”
12	Set 11 was limited to 12 September 2024
	Humans, no language restriction

Words written in *italic* were used as Medical Subject Headings (MESH) headings, the others were used as free text.

**Table 2.** **Summary of included studies on web-based psychosocial interventions in psychosis and/or schizophrenia**.

Study (Country)	Study design	Inclusion criteria	Primary and secondary outcomes	Characteristics of participants	Main findings
[3]	Mobile-based smartphone application intervention for adolescents with FEP in addition to usual treatment	14–19 yy	∙ Adherence treatment (MMAS)	25 randomized to intervention group (mobile app intervention) vs 25 randomized to control group (treatment as usual)	∙ Not available findings
(Spain; Barbeito *et al*., 2019)		Experience at least one positive psychotic symptom (delusions or hallucinations) before 19 years old in addition to 1 of the following DSM-5 diagnoses:			
			∙ Awareness illness (SUMD)		
	Pilot RCT, single-blind, single-centre		∙ Prognosis (SCPS)		
			∙ Quality of life (GAF, WHOQOL-BREF)		
		Schizophrenia, schizoaffective disorder, schizophreniform disorder, BD, MDD with psychotic features, brief psychotic disorder, or psychosis not otherwise specified.	∙ Symptomatology improvement (PANSS, STAI, HAM-D)		
[6]	Single-group follow-up pilot study using Horyzons, an online therapeutic platform that combines tailored, moderated social networking with psychoeducational resources to support recovery from early psychosis.	Young (15–25 yy) patients at their FEP in schizophreniform disorder (n = 4), schizophrenia (n = 3), schizoaffective or BD (n = 3), MDD with psychotic features (n = 4) and psychotic disorder not otherwise specified (n = 6) (DSM-IV).	∙ BPRS	20 young people enrolled in a specialist EPPIC coached to Horyzons functions and management of personal privacy without any time limitation in accessing the app over a period of 1 month	∙ Horyzons and the online social networking are considered safe and confidential by users.
(Australia; Gleeson *et al*., 2014)			∙ Safety, privacy and confidentiality		
					∙ All participants supported the role of moderators in maintaining a safe and welcoming environment.
	(4 weeks)				
				(20.3 ± 2.7)	
				(50% F, 50% M).	
[7]	Web-based psychoeducational program for caregivers of FEP patents	Caregivers of patients with FEP or psychosis at early stage in Hong Kong hospitals	∙ Modified version of the Scales for Perceived Usefulness and Perceived Ease of Use	809 caregivers registered iPEP as members	∙ A self-management approach to delivering psychoeducation to caregivers promotes flexibility and encourages active learning.
(China; Chan *et al*., 2016)				(67.4% F vs 31.6% M)	
				(44.2 ± 14.7 yy), of which 81 were randomly selected to participate in the interview.	
					∙ iPEP website is user-friendly.
[34]	RCT on web-based psychoeducation intervention	General population	∙ Impact on stereotypical perceptions of psychosis, such as dangerousness, unpredictability, blame, and pessimism about recovery.	178 participants received one of three psychoeducation texts	∙ Perceptions of dangerousness, unpredictability and anxiety towards people with schizophrenia were reduced.
(Germany; Schlier *et al*., 2016)				(i.e., medication, CBT or psychodynamic psychotherapy)	
					∙ Prognostic pessimism was reduced only after CBT program.
			∙ Impact on emotional reactions toward individuals with schizophrenia, including anxiety, anger, and sympathy.		
[35]	Observational study on video-based psychoeducation intervention	General population younger than 34 years old who are fluent in Chinese	∙ numbers of views	4935 views and 62 shares for a total viewing time of 35,614 minutes	∙ The most popular video was on FEP (62%), followed by schizophrenia (23.4%) and psychosis treatment (14.6%)
(China; Lam *et al*., 2017)			∙ total viewing time for each video		
	(12 months)				
			∙ demographic features of viewers	(49.9% F, 50.1% M)	
					∙ YouTube videos are attractive to the target audience.
[36]	Study on usefulness and performance of an Instagram advertisement promoting a YouTube video about FEP knowledge	Chinese-speaking population	∙ Metrics regarding the number of unique individuals reached and number of engagements after Facebook and Instagram advertisements were administered for 48 hours.	85 impressions on Facebook and 174 impressions on Instagram	∙ Instagram is non-inferior to Facebook in spreading psychoeducational material to general population.
(China; Lam & Woo, 2020)				24 engagements on Facebook and 42 engagements on Instagram	
[37]	Feasibility and efficacy RCT comparing a website psychoeducational program to caregivers’ homes via the Internet	Subjects with Schizophrenia or Schizoaffective Disorder aged 14 years or older (DSM-IV) with one or more psychiatric hospitalizations within the previous 2 years	∙ Socio-demographic data	A total of 30 patients with schizophrenia or schizoaffective disorder and 21 support persons were randomly assigned to either the telehealth intervention group (n = 16 patients with schizophrenia and 11 support persons) or the usual care group (n = 14 patients with schizophrenia and 10 support persons).	∙ Compared to control group, individuals with schizophrenia in the telehealth group reported significantly lower perceived stress and demonstrated a trend toward increased perceived social support.
(USA; Rotondi *et al*., 2005)			∙ Self-rated stress		
			∙ Social support		
	(SOAR)				
	(12 months)				
[38]	Qualitative study on an online selfhelp schizophrenia forum	Subjects with schizophrenia users of 12 international schizophrenia fora	∙ Analysis of online selfhelp coping strategies and usability and feasibility of online self-help schizophrenia forum	1200 postings analysed of 576 users	∙ The forum was predominantly used by affected individuals, few relatives or friends.
(Switzerland; Haker *et al*., 2005)					∙ The fields of interest regard daily issues of the illness (e.g., symptomatology and emotional involvement with the illness).
					∙ The most commonly used self-help mechanisms include disclosure and providing information; whilst emotional interaction, empathy and gratitude are rather rare.
[39]	Design and development of a computer-based self-management system for patients with schizophrenia spectrum psychosis	Subjects affected with psychosis spectrum disorder	∙ Ratings of levels of usability and feasibility of the online intervention pre- and post-intervention	316 inpatients	∙ Overall, the online intervention is considered useful, feasible and acceptable.
(Finland; Valimaki *et al*., 2008)				51 patient interviews	
				50 relative interviews	
				36 administrative personnel	
[40]	Adaptation of a program designed to leverage 7 Cups of Tea (7 Cups), an online platform of emotional support to schizophrenia patients	Outpatients with schizophrenia spectrum disorders (DSM-IV-TR)	∙ Attitudes and perceptions following chatting with listeners	10 schizophrenia outpatients	∙ Schizophrenia patients reported positive attitudes towards listeners trained to schizophrenia knowledge and considered the platform usable and helpful.
(USA; Baumel *et al*., 2016)			∙ Post-session open-ended comments		
			∙ Usability and usefulness ratings assessed on a 5-point Likert scale		
[41]	RCT of a pre-CBTp digital ‘informed choice’ intervention	Outpatient with a psychotic disorder diagnosis (ICD-10), aged 16–65	∙ Creating a new pre-CBTp intervention for informed decision-making aimed at addressing knowledge and attitudes.	40 psychotic patients and 40 clinicians working with people affected by psychosis	∙ Not available findings
(UK, Greenwood *et al*., 2018)					
[42]	Self-guided, recovery-focused online intervention for people with psychosis by using the SMART website	Affected by a nonorganic psychotic disorder (schizophrenia-related disorder [n = 60] or BD [n = 29] or MDD with psychotic features [n = 9] present within the past 2 years) (DSM-IV-TR)	∙ Engagement level	154 online enrolled, of which 113 were eligible to participate	∙ Both individual- and intervention-related factors influence engagement with a self-guided online intervention for psychosis
(Australia; Arnold *et al*., 2019)			∙ Socio-demographic information (i.e., age, gender, educational attainment, employment status)		
	Case-control study			(55% F vs 45% M)	
	(12 weeks)			(42 ± 11 yy)	
			∙ Frequency of Internet use by using a 6-item questionnaire	56 randomized to website only intervention (of which 51 completed) vs 57 randomized to + email intervention (of which 47 completed)	∙ Participants who received weekly, asynchronous emails from an online coach were more actively engaged and used the website more frequently than those who did not receive email support.
			∙ Recovery Style Questionnaire (RSQ)		
			∙ Modified version of the Autonomous and Controlled Motivations for Treatment Questionnaire (ACMTQ)		
[43]	Self-guided, web-based psychosocial intervention fostering personal recovery and self-management of mental health for people with psychosis	Diagnosis of a nonorganic psychotic disorder (schizophrenia-related disorder [n = 10] or BD [n = 4] or MDD with psychotic features [n = 3] present within the past 2 years) (DSM-IV-TR)	∙ Demographic information (i.e., age, gender, educational attainment, employment status)	98 online enrolled, of which 17 eligible to participate	∙ Challenges to using the website and factors supporting persistence are the two central themes related to participants’ engagement with the website.
(Australia; Arnold *et al*., 2020)				(65% F vs 35% M)	
				8 randomized to receive website only intervention vs 9 randomized to receive access to website + email intervention	
	Qualitative study		∙ Frequency of Internet use by using a 6-item questionnaire		
	(12 weeks)				
					∙ Amongst challenges have been included fluctuations in participants’ engagement; lack of time, space and resources and being overwhelmed by navigation.
			∙ Qualitative users’ experiences		
					∙ Amongst factors supporting persistence have been included taking a systematic approach, i.e., incorporating the SMART website into weekly routine activities.
[44]	Qualitative study on developing a blended (digital and in-person) intervention	Youths FEP	∙ Exploring young people’s views on a blended care model for FEP treatment design and delivery	10 participants	∙ Younger generations exhibit significant enthusiasm for hybrid models of mental health care.
(Australia; Valentine *et al*., 2020)				(aged 19 to 28)	
[45]	Qualitative study on social media-based mental health intervention	Youths FEP	∙ Young people’s subjective experiences	12 young people who utilized Horyzons were interviewed.	∙ Horyzon fostered a connection and an understanding amongst young people.
(Australia; Valentine *et al*., 2020)					∙ An increased sense of self-recognition and belonging over the long-term.
					∙ Factors such as social anxiety, feelings of paranoia, internalized stigma, a lack of perceived autonomy, and confusion about social norms online limited young people’s engagement with the platform.
[46]	Qualitative study of user needs and acceptability of a m-health intervention for schizophrenia patients	Treatment Resistant Schizophrenia patients	∙ Opinions of patients, caregivers, and healthcare providers regarding m-health services.	NA	∙ Webpages and online forums were considered appropriate platforms for reliable disease information and social support.
(Spain; Huerta-Ramos *et al*., 2017)					
[47]	Observational study on social media-based mental health intervention	Relatives of adolescents with a high clinical risk for psychosis	∙ Examine chat records to identify the specific needs and concerns of families during the initial phase of psychosis intervention	171 family members of 108 individuals identified as clinically high-risk for psychosis within the Shanghai at Risk for Psychosis research program	∙ Families of clinical high-risk individuals were highly involved, with primary concerns centering on functional recovery and medication.
(China; Zhang *et al*., 2018)					
[48]	Two-arm, unblended feasibility RCT on a smartphone-based intervention	Psychotic patients recruited by the Early Intervention Services in Psychosis (EIP)	∙ Feasibility and intervention engagement at 4 and 12 months.	40 participants were randomized with a 1:1 allocation to treatment as usual or treatment as usual plus access to ARIES.	∙ Recruitment and retention are feasible.
(UK; Steare *et al*., 2019)					
	(12 months)				
[49]	Pilot study RCT on a smartphone-based intervention	Psychotic patients recruited by the Early Intervention Services in Psychosis (EIP)	∙ Feasibility	40 participants recruited and assigned to Intervention group (n = 20) vs Treatment as usual group	∙ Not available findings
(UK; Steare *et al*., 2020)			∙ Relapse of psychosis		
	(12 months)		∙ Mental health and wellbeing		
			∙ Recovery		
			∙ Quality of life		
			∙ psychopathology		
[50]	RCT on a smartphone-based intervention	Psychotic disorders (DSM-5) recruited from early psychosis services	∙ Acceptability, feasibility and usability post-intervention and 3 months post-intervention	12 participants	∙ High levels of acceptability and feasibility
(Australia; Lim *et al*., 2020)	(6 weeks)				∙ Reduction of loneliness.
[51]	Qualitative study on smartphone-based ecological momentary assessment and intervention	Psychosis (DSM-5)	∙ Feasibility	12 participants	∙ Blended coping-focused therapy was perceived useful in providing experiences and increase the relationship with therapist.
(Australia; Moore *et al*., 2020)			∙ Experiences		
			∙ Perceptions		
[52]	Cross-sectional study on WeChat use and endorsement of WeChat-based mHealth amongst people living with schizophrenia	Schizophrenia patients (ICD-10) living with at least one family member and able to read and communicate	∙ WUIQ	Random sample of 400 Schizophrenia patients from 12 communities in Changsha City of Hunan Province (China)	∙ WeChat users are younger (*p* < 0.001), better educated (college and above; *p* < 0.001), and more likely to be employed (*p* = 0.001).
(China; Yu *et al*., 2020)			∙ BPRS		
			∙ GAF		
			∙ WHODAS 2.0		
			∙ PHQ-9		
			∙ GAD-7		∙ The most commonly endorsed WeChat-based mHealth program is psychoeducation (55.8%), followed by professional support (50.3%) and peer support (41.1%).
			∙ RAS		
			∙ WHOQOL-BREF		
					∙ WeChat users had lower scores in psychiatric symptoms (*p* = 0.030) and depression (*p* = 0.024) as well as higher scores in functioning (*p* < 0.001), recovery (*p* < 0.001) and quality of life (*p* = 0.002).
[53]	Feasibility study on a web-based application	Siblings and youth diagnosed with psychosis	∙ PSS	Of the 63 potential participants identified, 11 were ineligible, and 13 declined to participate. Thirty participants were enrolled and completed a baseline assessment, while 29 completed both baseline and at least one follow-up assessment component.	∙ The web-based application has been considered safe, acceptable, and feasible.
(Australia; Gleeson *et al*., 2017)			∙ DASS		
			∙ SPWB		
			∙ MOS-SSS		
[54]	Single-blind, parallel, online RCT on an online supported self-management toolkit	Relatives of people with psychosis or bipolar disorder	∙ Distress at 24 weeks (GHQ-28)	666 relatives	∙ Not available findings
(UK; Lobban *et al*., 2017)					
[55]	A theory-driven multiple case study design using a mixed methods for web-based interventions	Relatives of people with recent onset psychosis or bipolar disorder	∙ GHQ	NA	∙ Not available findings
(UK; Lobban *et al*., 2017)			∙ EQ-5D-5L		
			∙ eHEALS		
[56]	A single-blind, randomised controlled trial for web-based interventions	Relatives of people with recent onset psychosis or bipolar disorder	∙ GHQ	800 relatives of people with severe mental health problems	∙ The web-based application has been considered safe.
(UK; Lobban *et al*., 2020)					
[57]	A mixed-methods, theory-driven multiple case study investigation of a web-based peer-supported self-management intervention	Family members of individuals diagnosed with psychosis or bipolar disorder recruited by early intervention services in six English NHS mental health trusts	∙ Factors affecting implementation of REACT, including use of REACT and the impact of REACT in terms of relatives’ distress (GHQ-28) and carers’ wellbeing and support (Carer wellbeing and support scale questionnaire)	A total of 281 staff accounts and 159 relative accounts were included in the study. Of these, 129 staff and 23 relatives participated in qualitative interviews to share their experiences. Additionally, 132 relatives provided demographic information, 56 provided baseline data, 21 provided data at the 12-week follow-up, and 20 provided data at the 24-week follow-up.	∙ Participants, including staff and relatives, viewed REACT as a beneficial tool capable of enhancing support services and facilitating the achievement of clinical goals.
(UK; Lobban *et al*., 2020)					∙ Challenges to REACT’s implementation included heavy staff workloads, difficulties in prioritizing relative support, technical usability issues, incompatibility with existing Information technology (IT) systems and care processes, limited mobile access and forum participation, staff concerns about risk management, potential online harassment or job displacement, and uncertainty regarding REACT’s future.
[58]	Single-blind RCT comparing a resource plus REACT, a web-based, peer supported self-management intervention (REACT RCT)	Relatives of people with psychosis or BD	∙ Level of participants’ distress (GHQ-28 items)	800 relatives of people with SMI across the UK who were experiencing high levels of distress	∙ At 24 weeks, the mean scores for the GHQ-28 items reduced substantially across both arms over 24 weeks, with no significant difference between arms.
(UK; Lobban *et al*., 2020)				399 randomised to REACT plus resource directory vs 401 randomized to the resource directory only	
					∙ At 12 weeks, GHQ-28 was lower in the REACT arm compared to the resource directory only arm (*p* = 0.027).
[59]	A pilot feasibility randomized controlled trial (RCT) to co-develop and evaluate a novel e-learning resource designed to enhance the knowledge and attitudes of African-Caribbean families towards schizophrenia.	African-Caribbean families with schizophrenia or other non-affective psychosis (DSM-5) family members	∙ Relatives and carers of people with schizophrenia	40 aged ≥16 years randomized to receive intervention group (e-learning resource intervention) vs 40 aged ≥16 years randomized to control group (treatment as usual)	∙ Not available findings
(UK; Lemetyinen *et al*., 2018)			∙ Participant retention and attrition		
			∙ Improvement of knowledge and attitudes about schizophrenia and psychosis		
			∙ Acceptability of the intervention		
[60]	A web-based RCT to assess the efficacy of a structured e-health intervention development framework designed to support family members of individuals with psychosis in coping strategies (COPe-support).	Family members of individuals affected by psychosis	∙ Not provided	A multidisciplinary team, including public members, led a 1-year formative design and build process. This involved 4 co-production workshops and 2 rounds of focus groups with 24 carers (divided into 4 groups).	∙ Not available findings
(UK; Sin *et al*., 2020)					
[61]	A web-based RCT to assess the efficacy of a structured e-health intervention development framework designed to support family members of individuals with psychosis in coping strategies (COPe-support).	Family members of individuals affected by psychosis	∙ Effectiveness of COPe-support	204 families were randomly assigned to receive COPe-support, while the remaining 203 families were assigned to a control group. Access to the support materials was provided for 40 weeks, with participants encouraged to spend at least half an hour per week on the materials during the first 20 weeks.	∙ The use of COPe-support was not more effective than a passive online information resource.
(UK; Sin *et al*., 2022)					
[62]	A web-based RCT to assess the efficacy of a structured e-health intervention development framework designed to support family members of individuals with psychosis in coping strategies (COPe-support).	Family members of individuals affected by psychosis	∙ Explore carers’ experiences with COPe-support, including their perceived acceptability and its different components, how their engagement influenced their wellbeing and caregiving, and gather suggestions for improving the platform and its delivery to guide potential future broader implementation.	Interviews with 35 carers after their 8-month use of COPe-support as part of a web-based randomized controlled trial.	∙ All study participants reported a positive experience with COPe-support and endorsed its future implementation as a valuable additional resource for caregivers.
(UK; Batchelor *et al*., 2022)					
[63]	Mixed methods, multi-phase pilot study protocol for a digital intervention	FEP	∙ Feasibility and acceptability of a brief provider-led FAmily Motivational Engagement Strategy (FAMES)	An online survey was administered to 200 family members to evaluate the factors hindering and encouraging their participation in treatment.	∙ Not available findings
(USA; Oluwoye *et al*., 2020)					
				Five family members participated in a 3-month trial of the modified FAMES and implementation toolkit.	
				A 26-month stepped-wedge trial involving 50 family members	
[64]	A pilot study testing the Norwegian version of the REACT, a web-based, peer supported self-management intervention	Relatives of people with psychosis	∙ Baseline and 26-week assessments of distress and expressed emotion levels, measured using the Family Questionnaire and the Relatives Stress Scale.	Weekly family therapist support with 1 of 2 trained family therapists for 26 weeks were provided to 19 relatives.	∙ A marked reduction in both stress levels and emotional expression was observed among relatives of individuals diagnosed with psychosis.
(Norway; Romm *et al*., 2020)					
[65]	Convergent, parallel, mixed methods study on a Web-Based Psychoeducation intervention	Relatives of people with psychosis	∙ Feasibility of an online psychoeducational intervention for caregivers of individuals with schizophrenia spectrum disorders.	34 caregivers were recruited but 30 completed the second study.	∙ The intervention was considered feasible by almost of participants.
(Finland; Laine *et al*., 2021)					
[66]	Qualitative study on EOLAS, an online psychoeducation intervention	Patients with psychosis and their family member	∙ Feasibility, acceptability and usefulness of EOLAS-Online	16 patients and 21 family members participated in the online version of EOLAS program	∙ The overall results suggest that EOLAS-Online is feasible, acceptable, and useful in supporting participants on their recovery journeys.
(Ireland; O’Sullivan *et al*., 2023)					
[67]	Pilot study on an Internet-based psychoeducation and support programme	Relatives of people with early psychosis	∙ Exploring the feasibility and potential effectiveness of a novel German online psychoeducation and support program (ePSP)	25 families were eligible and consented to take part in the study.	∙ The interventions demonstrated significant positive impacts on primary outcome measures, including perceived stress and illness-related beliefs.
(Germany; Rus-Calafell *et al*., 2024)					
[68]	Comparative clinical trial comparing virtual reality (VR)-assisted therapy vs CBT	10 adult patients diagnosed with TRS (n = 8) or schizoaffective disorder (n = 2) who experienced persistent auditory verbal hallucinations	∙ Evaluate synergic effects of CBT for voices followed by VRT	10 patients assigned to the CBT+VR arm following completed follow-up after CBT treatment	∙ A combined intervention of VR and CBT demonstrated significant positive impacts on auditory verbal hallucinations, delusional beliefs related to voices, depressive symptoms, overall schizophrenia symptomatology, and quality of life.
(Italy; Dellazizzo *et al*., 2020)			∙ Overall severity of Auditory Verbal Hallucinations (PSYRATS-AH)		
				(20% F, 80% M)	
				(43.4 ± 14.6 yy)	
			∙ Assessing individual experiences with auditory verbal hallucinations, psychiatric symptoms, and overall well-being (BAVQ-R; BDI-II; PANSS; Q-LES-Q-SF)		
					∙ The combined CBT+VRT approach resulted in greater improvements in depressive symptoms and schizophrenia symptoms compared to either intervention delivered independently.
			∙ Evaluating patients’ perspectives on the individual therapies and the combination of CBT+VRT		
[69]	Quasi-experimental, feasibility and efficacy study of one-year, longitudinal, mirror-design study on a text-messaging intervention designed to identify early warning signs of relapse in patients with schizophrenia	Patients with schizophrenia (n = 29), schizoaffective disorder (n = 11) or acute polymorphic psychotic disorder with or without schizophrenia symptoms (n = 5)	∙ 10-item EWSQ	45 patients and 39 family members were enrolled.	∙ The number of hospitalizations decreased significantly by 60% (*p* < 0.001).
(Czech Republic; Španiel *et al*., 2008)					
				(40% F; 60% M)	
	(12 months)				
[70]	A quasi-experimental study examining the feasibility of an online group intervention for family members of individuals living with schizophrenia	Patients with schizophrenia and their relatives	∙ Symptomatology of patients (BPRS)	42 patients and relatives assigned to 26 online multifamily intervention group vs 16 assigned to TAU.	∙ Higher levels of satisfaction and attendance at the core online support session amongst participants who received online intervention compared to control group
(USA; Glynn *et al*., 2010)					
			∙ Relatives’ distress outcomes (somatic concerns and anxiety/depression subscales on the BSI)		
	(12 months)				
[71]	RCT, control-group study to assess efficacy of a psychoeducational website for patients with schizophrenia	Patients affected with schizophrenia and hospitalized more than once	∙ NA	28 patients enrolled were randomly assigned to 4 groups: 3 interventional groups (pharmacological treatment vs symptom management vs patients’ rights) or control group.	∙ Positive satisfaction level post web-based therapy
(Israel; Yakirevitch *et al*., 2010)					
					∙ Improvement of pharmacotherapy and symptom management after web-based treatment
	(2 weeks)				
[72]	RCT to investigate the impact of a web-based intervention on patient-clinician communication about evidence-based treatments for schizophrenia.	Patients with schizophrenia	∙ Communication indicators included visit length, the combined number of statements by the patient and clinician, and an index quantifying the extent of clinician verbal dominance.	26 patients randomized to intervention group vs 24 assigned to control group	∙ The use of internet-based resources enhanced communication and collaboration between individuals with schizophrenia and their healthcare providers, leading to more personalized treatment plans.
(USA; Steinwachs* et al*., 2011)					
[73]	Observational study aimed to collect qualitative and quantitative data from chart reviews in two psychiatric hospitals in Finland.	Inpatients with schizophrenia	∙ MMSE	93 patients were administered a web-based psychoeducation sessions conducted by 83 nurses in 9 inpatient units in Finland	∙ Patients were positively disposed towards psychoeducation, able to concentrate, to go locally through the webpages, to recall information that they had received in previous psychoeducational sessions.
(Finland; Anttila *et al*., 2012)			∙ GAF		
			∙ Vocational education level		
			∙ Socio-demographic features		
				(37.2 ± 12.2 yy)	
				(38% F; 62% M)	
[74]	Single group uncontrolled pilot study to evaluate the feasibility and efficacy of a text-messaging intervention	Patients affected by schizophrenia (n = 44) or schizoaffective disorder (n = 11)	∙ Daily ambulatory monitoring outcome assessment	55 patients recruited from outpatient settings	∙ A rise in social interactions was observed, accompanied by a reduction in the intensity of hallucinations. Real-time ecological momentary assessments demonstrated improved medication adherence.
(USA; Granholm *et al*., 2012)					
			∙ PANSS		
	(12 weeks)		∙ BDI-II		
			∙ ILSS		
			∙ ANARD		
					∙ No statistically significant enhancements were detected in depressive symptoms, psychotic manifestations, or independent living abilities at the follow-up evaluation.
[75]	Double-blind RCT to evaluate the effectiveness of a mobile phone-based short message service (SMS) platform in identifying early warning signs of relapse in individuals with schizophrenia	Patients with schizophrenia at increased risk for relapse	∙ NA	75 patients assigned to intervention group (ITAREPS) vs 71 subjects assigned to control group	∙ Patients who were administered the ITAREPS program displayed a nine-fold reduction in hospitalization compared to control group (*p* = 0.009).
(Czech Republic; Španiel *et al*., 2012)					
	(12 months)				∙ A significant difference in favour of intervention group was observed in the number of inpatient days and cost (*p* < 0.05).
[76]	Cross-sectional study to evaluate the usability of a web-based system integrating routine outcome monitoring and personalized advise in patients with schizophrenia spectrum disorders	Patients with schizophrenia, schizoaffective disorder, schizophreniform disorder, schizotypal disorder	∙ Learnability, efficiency, memorability, errors	15 patients were recruited using a snowball sampling technique from four mental health service providers in the Netherlands	∙ Schizophrenia patients can use the support system easily and they considered it meaningful and supportive.
(The Netherlands; van der Krieke *et al*., 2012)					
			∙ Satisfaction Questionnaire		
				(33% F, 66% M)	
				(42 yy).	
[77]	Single group uncontrolled pilot study to evaluate the feasibility, acceptability, safety and benefit of a web-based intervention	Young people with FEP, psychotic disorder or mood disorder with psychotic features (DSM-5), 15–25 aged, ≤6 months treatment with an antipsychotic medication, remission of positive symptoms of psychosis	∙ Participants’ usage (i.e., frequency, duration and patterns of use)	20 patients affected by FEP recruited from the Early Psychosis Prevention and Intervention Centre	∙ Integrated online therapy, social networking and peer and expert moderation in a real-world setting have been associated with a reduction in depression in FEP patients.
(Australia; Alvarez-Jimenez *et al*., 2013)					
			∙ Users’ experience (i.e., helpfulness, easy-to-use, attractiveness, safety, social interaction)		
	(4 weeks)			(20.3 ± 2.7)	
				(50% F, 50% M)	
					∙ 60% of patients described improvements in perceived social connectedness and 55% felt involved in their recovery process after the web-based intervention.
			∙ BPRS, CDSS, BAI		
[78]	Uncontrolled single group pilot study to evaluate the feasibility, acceptability and impact on hallucinations of a web-based CBT intervention	Adult participants (ages 18–65) diagnosed with schizophrenia, schizoaffective disorder, or other specified psychotic disorders according to DSM-IV criteria were included. Key inclusion criteria were: moderate to severe auditory hallucinations experienced within the previous week; no CBT received within the past 3 years; no hospitalizations or current suicidal ideation within the preceding month; stable antipsychotic medication regimen.	∙ PSYRATS	21 outpatients	∙ Significant improvements have been observed in hallucination severity and general psychopathology.
(USA; Gottlieb *et al*., 2013)			∙ BAVQ-R	(40.10 ± 13.63)	
			∙ WAIS		
			∙ Internet use and CBTp knowledge		
			∙ Program feasibility, acceptability and client satisfaction		
[79]	Pilot study on a web-based intervention	Persisting psychotic disorders	∙ Feasibility and acceptability of intervention	10 participants with persisting psychotic disorders	∙ Personal recovery had improved post-intervention.
(Australia; Thomas *et al*., 2016)					
[80]	Rater-blinded RCT on a web-based intervention	Patients with a primary diagnosis of schizophrenia spectrum disorders or those with mood disorders who have experienced psychotic symptoms	∙ Personal recovery measured using the Process of Recovery Questionnaire (QPR) at 3, 6 and 9 months post-baseline.	148 schizophrenia-related disorder or mood disorder with a history of psychosis were recruited.	∙ Findings still not available.
(Australia; Thomas *et al*., 2016)					
			∙ Positive and negative symptomatology (PANSS), subjective experiences of psychosis		
			∙ Emotional well-being, quality of life and resource use		
[81]	Pilot study of a RCT examining feasibility and effectiveness of an online social cognitive training	Schizophrenia diagnosis (DSM-IV-TR)	∙ Feasibility of the training	17 schizophrenia patients completed 24 hours of online SocialVille game play either from home or at a clinic, over a 6–10-week period vs 17 matched healthy controls.	∙ Participants with schizophrenia reported medium to high levels of satisfaction, enjoyment, and usability regarding the SocialVille platform.
(USA; Nahum *et al*., 2014)			∙ Gains on the SocialVille exercises related to control group		
	(10 weeks)				
			∙ Variation on measures of social cognition, social functioning, global functioning and motivation		
					∙ Significant and large improvements on the speeded SocialVille tasks have been reported in schizophrenia groups vs control group.
					∙ Small to moderate improvements on the working memory tasks have been observed in schizophrenia group vs control group.
					∙ After completing the training program, individuals with schizophrenia exhibited performance on the SocialVille tasks that was comparable to the baseline performance of the healthy control group.
					∙ Schizophrenia patients exhibited enhanced performance across a range of standardized assessments evaluating social cognitive abilities, social functioning, and motivational levels.
[82]	RCT on a web-based intervention	Schizophrenia patients (DSM-IV-TR)	∙ Ekman 60 Faces Test	12 patients were assigned to the intervention group vs 9 patients were assigned to control group (occupational therapy and leisure group).	∙ The intervention group reported efficacy in improving emotion recognition and statistically significant improvements (*p* < 0.05) for the Ekman 60 Faces Test, theory of mind (Hinting Task, Faux Pas, Happé) and AIHQ, after the treatment.
(Spain; Vázquez-Campo *et al*., 2016)			∙ Theory of mind (Hinting Task, Recognition of Faux Pas, Strange Stories of Happé)		
			∙ AIHQ		
			∙ MSCEIT		
			∙ PANSS		
			∙ WAIS		
[83]	Single-blind RCT of Horyzons, an online treatment application	FEP from Early Psychosis Prevention and Intervention Centre	∙ Social functioning, rate of hospital admissions, cost-effectiveness, vocational status, depression, social support, loneliness, self-esteem, self-efficacy, anxiety, psychological wellbeing, satisfaction with life, quality of life positive and negative psychotic symptoms and substance use at 6, 12 and 18 months of follow-up	170 young people with FEP were assigned to Horyzons plus treatment as usual or treatment as usual alone.	∙ Findings still not available.
(Australia; Alvarez-Jimenez *et al*., 2019)					
	(18 months)				
[84]	Design of a web-based intervention	Youth with FEP	NA	NA	∙ Findings still not available.
(Australia; McEnery *et al*., 2019)					
[85]	Pilot study of web-based intervention	FEP with a sub-threshold clinical score >30 on the Social Interaction Anxiety Scale	∙ SIAS	10 participants (aged 17–26)	∙ 7/10 participants completed 8 modules or more of EMBRACE program.
(Australia; McEnery *et al*., 2021)			∙ LSAS		
					∙ All participants reported a positive and favorable experience with the intervention, perceiving it as safe and recommending it as a potential resource for individuals experiencing social anxiety.
					∙ Statistically significant improvements were found in SIAS (*p* = 0.0005) and LSAS (*p* = 0.002).
					∙ No statistically significant differences were found for depressive or loneliness symptomatology.
[86]	Pilot study of a home-delivered web-based Cognitive Remediation intervention	FEP outpatients	∙ CGI	17 participants were assigned to a cognitive training protocol.	∙ Overall participants’ evaluation is positive.
(Portugal; Moura *et al*., 2019)			∙ PANSS		
			∙ Sustained attention		∙ A statistically significant enhancement was observed in both sustained attention (*p* = 0.020) and verbal memory (*p* = 0.018).
	(6 months)		∙ Verbal memory		
					∙ A statistically significant reduction in negative symptoms was accompanied by an improvement in CGI score (*p* = 0.009).
[87]	RCT on a web-based intervention	FEP	∙ Feasibility	26 participants recruited, of which 15 clinicians (aged 26–56) and 11 FEP (aged 19–37).	∙ Both patients and clinicians expressed positive feedback regarding the strengths-based framework and the integration of social media functionalities within the Horyzons program.
(Canada; Lal *et al*., 2020)			∙ Adaptation and improvements to the original Horyzons		
					∙ Several participants raised concerns regarding the practical aspects of implementing the program, particularly in terms of resource availability. These concerns included the need for adequate support for site moderation, crisis management, and sufficient internet connectivity, especially in areas with limited access.
[88]	Pre-post mixed methods (qualitative-quantitative convergent) design on a web-based intervention.	FEP	∙ Acceptability	Baseline data was collected from 23 participants, while follow-up data was obtained from 20 participants after an 8-week intervention period.	∙ Feasible, acceptable and potentially effective
(Canada; Lal *et al*., 2021)			∙ Safety		
			∙ Potential efficacy		
[89]	Uncontrolled single-group, pre-post (8 weeks), mixed methods study on a web-based intervention	FEP	∙ Safety	Baseline data was collected from 23 participants, while follow-up data was obtained from 20 participants after an 8-week intervention period.	∙ Social functioning remained largely stable, and there was no evidence of clinical deterioration based on the Clinical Global Impression Scale.
(Canada; Lal *et al*., 2023)			∙ Effects on relapses and social functioning		
					∙ Feasible, safe and acceptable
[90]	RCT on a web-based intervention	Psychotic patients with sleep problems and worrying thoughts	∙ Paranoia, auditory verbal hallucinations and theory-driven precursors worrying, negative affect, self-esteem, self-reported cognitive biases and quality of sleep	124 participants	∙ Findings suggest that elevated levels of worry and disrupted sleep patterns may serve as potential predictors of increased momentary psychotic symptoms during interventions.
(Switzerland; Lüdtke *et al*., 2021)	(8 weeks)				
[91]	Secondary analysis of a RCT evaluating a psychological web-based intervention for psychosis	Psychotic patients	∙ Hallucinations	16 participants	∙ Post-assessment findings revealed significantly higher levels of mindfulness (*p* = 0.015) and significantly lower levels of hallucinations (*p* = 0.001) in the intervention group compared to the control group.
(Switzerland; Lüdtke *et al*., 2020)			∙ Mindfulness abilities		
			∙ Distress levels associated with hallucinations		
					∙ No significant differences on distress by voices between two groups.
[92]	RCT on a web-based intervention	FEP with a diagnosis of schizophrenia spectrum disorder (DSM-5)	∙ Experiences and perceptions by users	26 participants	∙ Horyzons is feasible and very well tolerated.
(USA; Ludwig *et al*., 2021)	(12 weeks)				
			∙ Psychotic symptomatology and loneliness		∙ Greater improvements in psychosis-related symptoms, followed by self-reported experience of negative emotions, depressive symptomatology and loneliness following intervention.
[93]	Double-blind, controlled, RCT on a web-based intervention	Schizophrenia outpatients (DSM-5)	∙ Social cognitive composite	55 schizophrenia patients assigned to intervention group (SocialVille) vs 53 assigned to active control (computer games)	∙ Statistically significant improvement of social cognitive composite (*p* < 0.001) in intervention group compared to control group.
(USA; Nahum *et al*., 2021)			∙ Functional capacity outcome (UCSD Performance-based Skills Assessment, UPSA-2)		
			∙ Virtual functional capacity measure (VRFCAT)		∙ Improvements (not significant) in social functioning, virtual functional capacity measure and motivational subscale in intervention group.
			∙ Social functioning		
			∙ Quality of life and motivation		
[94]	RCT on a computer-based recovery-oriented intervention	FEP (aged 12–25)	∙ Lived experienced	10 young adults (4 females, 6 males) aged 18 to 31 years old (M = 23.10, Standard Deviation = 3.84) who experienced psychosis	∙ Not available findings
(Australia; Peck *et al*., 2020)			∙ Personally recovery		
[95]	RCT, single blind on a SMS (text) messaging intervention	FEP (16–29 yy)	∙ Attendance at the first consultation appointment within 30 days of study enrolment	186 participants referred by the emergency department to early psychosis services recruited for a trial of a two-way intervention involving reminders, psychoeducation and check-ins delivered by SMS	∙ Not available findings
(Canada; Polillo *et al*., 2020)					
	(24 months)				
			∙ Routine clinical measures (BPRS, CGI, SES)		
[96]	A pilot single-blind, control-group, RCT of a CACR program to help young people with psychosis to restore cognitive function	Young people (aged 15–28) diagnosed with psychosis or schizophrenia and who were referred to occupational therapy service	∙ Cognitive functioning (i.e., speed of processing, attention/vigilance, working memory, verbal learning, visual learning, and reasoning and problem-solving) (MATRICS Consensus Cognitive Battery)	40 young people with psychosis or schizophrenia	∙ The CACR program demonstrated efficacy in enhancing cognitive function across various domains, including verbal learning and speed of processing, when compared to a control group.
(China; Siu *et al*., 2021)					
				(21.9 ± 3.4)	
				(54.5%)	
				20 randomized to intervention program vs 20 randomized to treatment as usual	
			∙ Mental wellbeing (PANSS; Chinese Short Warwick-Edinburgh Mental Wellbeing Scale)		∙ The intervention group experienced significant positive changes in mental well-being and perceived occupational competence compared to the control group.
			∙ Perceived competence in occupational functioning (OSA)		
			∙ Engagement in occupational roles like worker, student, trainee or homemaking		∙ No significant variations in symptoms when we compared the control vs interventional group, despite the interventional group had a significant reduction in negative symptomatology over pre-, post- and follow-up.
[97]	RCT on an internet-based intervention	Psychotic patients	∙ PANSS	NA	∙ Not available findings
(Switzerland; Rüegg *et al*., 2018)					
[98]	Feasibility study on a web-based application	Carers of youth affected by FEP, depression and anxiety	∙ Acceptability and safety of the online program	63 enrolled, of which 30 were eligible to participate	∙ A statistically significant reduction in self-reported levels of perceived stress (*p* = 0.003) amongst caregivers of patients with SMI
(Australia; Gleeson *et al*., 2017)					
			∙ Baseline scores on measures of stress, depression, anxiety, psychological well-being, and social support (PSS, DASS, SPWB, MOS-SSS) were compared to scores obtained at a 3-month follow-up assessment.	(86% F, 14% M)	
				(47.8 ± 6.4 yy).	
[99]	Nonrandomized feasibility and usability study on web-based virtual learning environment with siblings of individuals diagnosed with psychosis	Siblings of patients with psychosis	∙ Socio-demographic data	20 siblings, of which 17 completed the online evaluation after using the intervention.	∙ Overall, the online intervention is considered useful, feasible and acceptable to improve awareness and information as well as peer-support intervention to promote wellbeing and coping strategies to manage patients with psychosis.
(UK; Sin *et al*., 2019)			∙ Ratings of levels of usability and feasibility of the online intervention pre- and post-intervention		
	(4 weeks)				

AIHQ, Ambiguous Intentions Hostility Questionnaire; ANARD, American National Adult Reading Test; ARIES, App to support Recovery in Early Intervention Services; BAI, Beck Anxiety Disorder; BAVQ-R, Belief about Voices Questionnaire-revised; BD, bipolar disorder; BDI-II, Beck Depression Inventory-II; BPRS, Brief Psychiatric Rating Scale; BSI, Brief Symptom Inventory; CACR, computer-assisted cognitive remediation; CBT, cognitive behavioural therapy; CBTp, cognitive behavioural therapy for psychosis; CDSS, Clinician-rated Calgary Depression Scale for Schizophrenia; CGI, Clinical Global Impression; DASS, Depression, Anxiety Stress Scale; DSM, Diagnostic Statistical Manual; eHEALS, eHealth literacy scale; EPPIC, Early Psychosis Prevention and Intervention Centre; EQ-5D-5L, 5-level EQ-5D version; EWSQ, Early Warning Signs Questionnaire; FEP, first episode psychosis; GAD-7, Generalized Anxiety Disorder Scale-7; GAF, Global Assessment of Functioning; GHQ, General Health Questionnaire; HAM-D, Hamilton Rating Scale for Depression; iPEP, psychosis psychoeducational program for caregivers; ITAREPS, Information Technology Aided Relapse Prevention Programme in Schizophrenia; F, female; M, male; ILSS, Independent Living Skills Survey; LSAS, Leibowitz Social Anxiety Scale; MDD, Major Depressive Disorder; MMAS, Morinsky Medication Adherence Scale; MMSE, Mini Mental State Examination; MOS-SSS, Medical Outcomes Study, Social Support Survey; MSCEIT, Mayer-Salovey-Caruso Emotional Intelligence Test; NA, not applicable; NHS, National Health Service; OSA, Occupational Self-Assessment; *p*, *p*-value; PANSS, Positive and Negative Symptom Severity; PHQ-9, Patient Health Questionnaire; PSS, Perceived Stress Scale; PSYRATS, Psychotic Symptom Rating Scales; RCT, Randomized Clinical Trial; RAS, Recovery Assessment Scale; REACT, Relatives Education and Coping Toolkit; Q-LES-Q-SF, Quality of Life Enjoyment and Satisfaction Questionnaire - Short Form; SCPS, Strauss and Carpenter Prognostic Scale; SES, Service Engagement Scale; SIAS, Social Interaction Anxiety Scale; SMART, Self-MAnagement and Recovery Technology; SOAR, Schizophrenia Online Access to Resources; SPWB, Scales of Psychological Wellbeing; STAI, State-Trait Anxiety Inventory; SUMD, Scale to Assess Unawareness of Mental Disorder; yy, years; TAU, treatment as usual; TRS, treatment-resistant schizophrenia; UCSD, University of California, San Diego Performance; UPSA, UCSD Performance-based Skills Assessment; VRFCAT, Virtual Reality Functional Capacity Assessment Tool; VRT, virtual-reality-assisted therapy; WAIS, Wechsler Adult Intelligence Scale; WHODAS 2.0., 12-item World Health Organization Disability Assessment Schedule 2.0.; WHOQOL-BREF, World Health Organization Quality of Life Brief Scale; WUIQ, WeChat Use intensity questionnaire; SMI, severe mental illness; COPe-support, Carers fOr People with Psychosis e-support; DSM-IV-TR, Diagnostic and Statistical Manual of Mental Disorders-IV-TR; ICD-10, International Classification of Diseases-10.

**Table 3.** **Characteristics of the digital interventions included in this review**.

Study and type of intervention	Characteristics of the intervention
[3]	A mobile app-based intervention to improve community functioning, quality of life, illness awareness and treatment adherence in adolescents with FEP. The psychotherapy app programme is structured around five distinct modules: a psychoeducational module, a module on recognition of symptoms and prevention of relapses, a problem-solving module, a mindfulness module, contact wall module.
Horyzons	Horyzons is a moderated online social therapy (MOST), integrating interactive psychoeducation and therapeutic online social networking by involving online moderators, specifically designed to assist young FEP patients. It combines online therapy modules with moderated social networking features, including psychoeducation, stigma, early relapse warning signs, depression, social anxiety, stress, and the recognition and utilization of personal strengths.
[6, 45, 77, 83, 87, 88, 89, 92]	
[7]	A web-based psychosis psychoeducational program for caregivers (iPEP) comprise web-based psychoeducational materials to facilitate self-learning of caregivers of patients with psychosis, by also using an interactive online forum for sharing and dissemination amongst caregivers and communications with mental health professionals as well as face-to-face group-based psychoeducation to provide an offline interactive platform among the caregivers.
[34]	Three psychoeducational interventions focuses on medication, CBT and psychodynamic psychotherapy in reducing stigmatizing perceptions towards people with schizophrenia amongst the general population.
[35]	Three YouTube educational 48-min-long videos related to psychosis.
[36]	Instagram advertisement publicizing a YouTube video on FEP knowledge for targeted population aged 18–34 years.
SOAR	A psychoeducational program (named ‘Schizophrenia Guide software’) delivered via telemental health addressed to schizophrenia subjects and their caregivers. The software provides the following services: (a) three online therapy groups; (b) the possibility to consult with the project’s experts and receive a response; (c) a collection of previously asked and answered questions; (d) community events and news stories centred on mental health topics; (e) educational reading resources.
[37]	
[38]	An online self-help forum for individuals with schizophrenia and their parents with useful tool to cope with alienation and isolation.
Mieli.Net portal	A computer-based support system specifically designed for patients with schizophrenia spectrum psychosis. It offers information about the disease, a channel for peer support for patients, a tool for counselling and a chat for the interactions with doctors.
[39]	
7 Cups of Tea (7 Cups)	An online anonymous portal that connects individuals seeking emotional support with trained, empathetic listeners from around the globe as an adjunct to treatment of people with schizophrenia spectrum disorders.
[40]	
[41]	A pre-CBT digital psychoeducation intervention composed by a website containing information, interactive elements and animated stories, as well as an interactive goals section aimed to encourage motivation.
SMART website	The Self-MAnagement and Recovery Technology (SMART) website is a self-guided, recovery-focused online intervention for people with psychosis. The modules of the SMART website are the following ones: (a) recovery (i.e., promoting hope); (b) stress management (i.e., common stressors, approaches on how to cope with them); (c) health (i.e., self-management, medication and sleep); (d) me (i.e., identity, personal strengths and stigma); (e) relationships (i.e., interpersonal relationships and social competence); and, (f) life (i.e., values and goals). Each module offers videos of individuals who experienced psychosis discussing their individual experiences and feelings regarding specific topics. Moreover, the SMART website contains exercises pertaining to module content, social networking features (i.e., forum, opportunity to comment publicly and interact with other users) and self-management tools (i.e., charts for stress, mood and sleep). In addition to the SMART website, patients may receive weekly, asynchronous emails from an online coach over 12-weeks with the aim to encourage patients to work through the website content.
[42, 43, 80]	
[44]	A combined approach incorporating both digital and face-to-face interventions to enhance care of patients affected by FEP.
m-RESIST	Mobile Therapeutic Attention for Patients with Treatment Resistant Schizophrenia (m-RESIST) is a web-delivered intervention, composed by the following modules: psycho-education, monitoring, treatment, and illness self-management.
[46]	
[47]	A family-focused WeChat-based online social networking intervention targeting functional recovery and addressed to family members of youths at clinical high risk of psychosis.
My Journey 3	A self-management smartphone app intervention for adults receiving Early Intervention Services in Psychosis (EIP) services to support recovery. Through My Journey 3, the patients receive information regarding psychosis, mental health and mental health services. It offers a self-monitoring tool, a symptom tracker and a pill tracker.
[48, 49]	
+Connect	A digital smartphone app designed to address loneliness among young individuals experiencing psychosis. It offers videos regarding the experience of illness and how to cope with emotions.
[50]	
EMA/I	A novel mobile intervention that integrates real-time assessment and personalized support through a smartphone application, aiming to enhance coping mechanisms in individuals experiencing persistent auditory verbal hallucinations.
[51]	
[52]	A WeChat-based mHealth program to support global recovery while enhancing the physical and mental wellbeing of individuals with schizophrenia.
[53, 98]	A web-based application integrates several key components: online psychoeducation and interactive therapy (divided into specific thematic pathways which are further separated into individual ‘steps’), expert-moderated social networking (via a ‘café’), and peer moderation.
REACT	An online program designed to enhance self-management skills among family members of individuals living with psychosis or bipolar disorder. This program incorporates peer support and provides access to educational resources and a comprehensive toolkit of coping strategies.
[54, 55, 56, 57, 58, 64]	
CaS-PER	A web-based e-learning resource was developed to improve the understanding of schizophrenia and related psychotic disorders among family members and caregivers of individuals of African-Caribbean descent, called Culturally Appropriate Schizophrenia Psychological Education Resource (CaS-PER).
[59]	
COPe-support	An e-health intervention named COPe-support (Carers fOr People with Psychosis e-support), in promoting caregivers’ health outcomes, information and psychosocial support for caregivers of subjects affected with psychosis through the Internet, promoting flexible access and individualized choice.
[60, 61, 62, 99]	
FAMES	An iterative mixed methods for caregivers and family members of patients with FEP.
[63]	
[65]	A web-based psychoeducation course to provide information and peer support for caregivers of individuals with psychotic disorders. The course lasted 8 weeks and included 6 modules, each focused on a different theme: orientation, daily life, mental illness, patient and caregiver rights, treatment and wellbeing.
EOLAS	A recovery-oriented psychoeducation programme for psychosis designed for patients and their families. The structured programs consist of eight weekly group sessions, each lasting 90 minutes. These sessions cover topics such as psychosis, biopsychosocial treatment options, accessing services and support, as well as addressing stigma and promoting self-advocacy.
[66]	
ePSP	A German-moderated online psychoeducation and support programme (ePSP) for family members of individuals experiencing early stages of psychosis. This intervention has been set up as an online self-learning course on the platform Moodle, with content evidence-based about psychological interventions, peer-support principles in the context of family interventions for psychosis, self-care interventions, and communication and problem-solving skills learning programmes. It comprises 5 modules: “What is Psychosis?”, “Treatment & Crisis”, “Communication & Emotion”, “Self-Compassion”, “Health Services”.
[67]	
[68]	CBT for voices followed by virtual-reality-assisted therapy (VRT) for patients with treatment-resistant schizophrenia or schizoaffective disorder.
ITAREPS	A text-messaging intervention, designed to identify early warning signs of relapse in patients with schizophrenia and named Information Technology Aided Relapse Prevention Programme in Schizophrenia (“ITAREPS”).
[69, 75]	
[70]	An online multifamily group educational program with a cohort of patients affected by schizophrenia and their relatives.
[71]	A web-based psychoeducation in a sample of patients affected with chronic schizophrenia.
[72]	A web-based tool to support patients with schizophrenia by facilitating informed discussions with healthcare providers regarding evidence-supported treatment options. It is composed by an interactive website with information on six domains (i.e., referrals, quality of life, medications, side effects, employment and family support).
[73]	A web-based psychoeducation intervention consisting of six psychoeducation sessions which were used over a period lasting between 1 and 70 days and took 10–360 minutes per patient. Psychoeducation sessions dealt with mental illness, treatment, wellbeing, patients’ rights and daily life.
MATS	A mobile phone-based text message intervention called Mobile Assessment and Treatment for Schizophrenia (MATS) which provided health promoting behaviours by using CBT techniques.
[74]	
[76]	A web-based support system which allows routine outcome monitoring more accessible to patients with schizophrenia, by showing a valuable potential of the tool in improving routine outcome monitoring practice for psychotic patients.
[78]	A web-based CBT for auditory hallucinations in subjects with psychosis.
[79]	A website to be used on a tablet computer by mental health workers to structure therapeutic sessions about personal recovery along a 8-session low intensity intervention addressed to individuals with persisting psychotic disorders.
SocialVille	A neuroplasticity-based online social cognitive training program (named “SocialVille”) addressed to young people with schizophrenia.
[81, 93]	
e-Motional Training	A novel web-based training program designed to enhance social cognition in people diagnosed with schizophrenia.
[82]	
EMBRACE	A moderated online intervention designed to address social anxiety in FEP individuals.
[84, 85]	
[86]	A home-delivered web-based intervention of cognitive remediation targeted to FEP individuals. It has positive effect on sustained attention, verbal memory, negative and positive symptomatology.
EviBaS	EviBaS is an online intervention for individuals with psychosis that incorporates CBT principles. The program consists of 11 modules: an introductory module, a relapse prevention module, and nine modules addressing specific psychotic symptoms and associated challenges. These challenges include persecutory delusions, auditory hallucinations, excessive worry, diminished mindfulness, impaired social skills, low self-esteem, depressive symptoms, sleep disturbances, and cognitive biases.
[90, 91, 97]	
[94]	A web-based digital tool was created for peer learning activities, specifically designed for tablet devices. This resource comprised fourteen videos organized into six overarching thematic categories (i.e., My Journey, Self-Care, Connections, My Identity, Life, and Mental Health for FEP).
[95]	A web-based intervention using short message service (SMS) was designed to improve transitions from the emergency department to evidence-based early psychosis intervention services.
[96]	A standardized computer-assisted cognitive remediation program administered to young people affected with psychosis or schizophrenia, by comparing outcomes following the intervention program.

EMA/I, ecological momentary assessment and intervention.

### Studies on Users’ Engagement and Experiences

A feasibility study was drafted to evaluate the preliminary efficacy of a psychoeducational program (named ‘Schizophrenia Guide software’) delivered via telemental health addressed to schizophrenia subjects and their caregivers [37]. The software provides the following services: (a) three online therapy groups; (b) the possibility to consult with the project’s experts and receive a response; (c) a collection of previously asked and answered questions; (d) community events and news stories centred on mental health topics; (e) educational reading resources [37]. Following 3 months of intervention, schizophrenia individuals who were involved in the telehealth intervention displayed a higher reduction in stress levels in comparison to those in the usual care group and a trend to greater perceived social support [37].

Haker *et al*. [38] analysed experiences and perceptions collected by online self-help for individuals with schizophrenia and their parents by suggesting that online forums may be a useful tool to cope with alienation and isolation for patients with schizophrenia. A patient-centred, health-oriented, supportive self-care and self-management, computer-based support system was specifically designed for patients with schizophrenia spectrum psychosis by overly demonstrating that patients considered the web-based intervention useful and acceptable [39].

Baumel *et al*. [40] adapted a peer-based online emotional support program (designed to leverage the platform 7 Cups of Tea, 7COT, an online anonymous portal that connects individuals seeking emotional support with trained, empathetic listeners from around the globe) as an adjunct to treatment of people with schizophrenia spectrum disorders, by demonstrating an overall positive attitude and perception amongst schizophrenia participants.

A two-arm, feasibility randomized controlled trial (RCT) of a digital ‘informed-choice’ decision aid for implementing CBTp was developed to evaluate a pre-CBT digital psychoeducation intervention developed to address identified knowledge and attitudes to uptake and implement CBTp [41]. The study is still in the recruitment stage and no findings are available. The pre-CBT digital psychoeducation intervention consists of a website containing interactive elements, information and animated stories, and an interactive goals section to encourage motivation [41].

A study carried out within the Self-MAnagement and Recovery Technology (SMART) research program in Australia evaluated psychological, demographic and treatment variables which may predict engagement with a self-guided, recovery-focused online intervention for people with psychosis [42]. The modules of the SMART website are the following ones: (a) recovery (i.e., promoting hope); (b) stress management (i.e., common stressors, approaches on how to cope with them); (c) health (i.e., self-management, medication and sleep); (d) me (i.e., identity, personal strengths and stigma); (e) relationships (i.e., interpersonal relationships and social competence); and, (f) life (i.e., values and goals). Each module offers videos of individuals who experienced psychosis discussing their individual experiences and feelings regarding specific topics. Moreover, the SMART website contains exercises pertaining to module content, social networking features (i.e., forum, opportunity to comment publicly and interact with other users) and self-management tools (i.e., charts for stress, mood and sleep). In addition to the SMART website, patients may receive every week, asynchronous emails from an online coach over 12-weeks with the aim of encouraging patients to work through the website content. The study compared the group engaged only with the website versus the group engaged with the website and an email support service by reporting a substantially increased impact on engagement amongst patients receiving emails (more than 40%) compared to those accessing the website independently [42]. A further study, embedded with the SMART project, consisted of a qualitative investigation to explore users’ experiences and perspectives about variables influencing the level of engagement and adherence with a web-based psychosocial intervention for individuals with psychosis [43]. Authors reported fluctuations in mental health and psychosocial difficulties among the most significant challenges to be overcome in order to be more engaged and persistent in web-based psychosocial interventions [43].

A qualitative study carried out by Valentine *et al*. [44] sought to gather insights from young people on the development and execution of a blended care model for treating FEP, and found that this approach was associated with more positive attitudes, increased accessibility, continuity and consolidation between patients and clinicians. In a further qualitative analysis of young people’s experiences of a long-term social media-based intervention for FEP designed to address social functioning, the same group reported overly positive attitudes even though barriers that could substantively limit their ability to use the platform (e.g., social anxiety, paranoia) were also described [45].

### Studies on Mobile App-Based Psychosocial Interventions

A moderated online social therapy (MOST), integrating interactive psychoeducation and therapeutic online social networking by involving online moderators, has been applied within an application named ‘Horyzons’, specifically designed to assist young FEP patients [6]. The application combines online therapy modules with moderated social networking features, including psychoeducation, stigma, early relapse warning signs, depression, social anxiety, stress, and the recognition and utilization of personal strengths. The study was carried out at the Early Psychosis Prevention and Intervention Centre (EPPIC) in Australia and authors concluded that the MOST model offers a secure and effective approach for engaging young people in long-term psychosocial interventions with the final aim of reducing relapse risk and facilitating functional recovery [6]. Another study evaluated the efficacy and user acceptability of an m-health intervention called Mobile Therapeutic Attention for Patients with Treatment Resistant Schizophrenia (m-RESIST), reporting a positive acceptance and usefulness by patients, informal carers and clinicians [46].

A pilot study proposed an innovative protocol to evaluate the effectiveness of a mobile app-based intervention to improve community functioning, quality of life, illness awareness and treatment adherence in adolescents with FEP as a complement to their usual treatment [3]. The mobile phone psychotherapy app was composed of five modules: (a) psychoeducation (12 sessions); (b) recognition of symptoms and relapse prevention; (c) problem-solving based on McFarlane’s Multiple Family Therapy model [100, 101, 102]; (d) mindfulness (3 recordings) [103]; (e) contact wall [3]. A protocol and a feasibility study exploring a self-management smartphone app intervention for adults receiving Early Intervention Services in Psychosis (EIP) services to support recovery named App to support Recovery in Early Intervention Services (ARIES) were published by Steare *et al*. [48, 49]. A pilot study evaluated the effectiveness of a digital smartphone app called “+Connect”, designed to address loneliness among young individuals experiencing psychosis, which demonstrated high acceptability and feasibility [50]. A new smartphone-based intervention combining ecological momentary assessment and intervention (EMA/I) was developed to enhance coping strategies for individuals with persistent auditory verbal hallucinations. When compared to four in-person therapy sessions, participants reported that the EMA/I technology provided a more precise reflection of their experiences [51]. A Chinese study examined WeChat use, preferences for WeChat-based mHealth programs and health outcomes of WeChat users in an urban community sample of schizophrenia patients; authors report that WeChat-based mHealth interventions represent an empowering tool to provide cost-effective interventions that support global recovery while enhancing the physical and mental wellbeing of individuals with schizophrenia [52]. WeChat is the most prevalent mobile app in Chinese, which literally means ‘micro message’ with characteristics similar to WhatsApp for message release and Facebook’s newsfeed by allowing members to post pictures, text messages, emojis, webpages and even short videos to Moments and give and get comments. The findings showed that the WeChat use rate was 40.8% in the sample of schizophrenia patients, with 30.7% of the patients who had more than 50 WeChat friends and nearly half spent more than half an hour on WeChat by declaring a willingness to participate in any kind of WeChat-based mHealth program in around 80.4% of the sample [52]. A family-focused WeChat-based online social networking intervention targeting functional recovery and addressed to family members of youths at clinical high risk of psychosis demonstrated a high involvement of families of clinical high-risk individuals for psychosis [47].

### Studies on Web-Based Psychoeducational Interventions for Family/Caregivers of Patients with Schizophrenia and Psychosis 

A multifamily psychoeducational intervention designed for schizophrenia individuals and their informal supports (i.e., family and friends) reported a statistically significant reduction in positive symptomatology and considerable and significant improvements in knowledge of schizophrenia compared to treatment as usual group [104]. A non-randomized usability study with siblings of individuals diagnosed with psychosis was carried out to collect feedback from participants about the ease of usage as well as the perceived usefulness and acceptability of the digital intervention [105]. Overall, siblings of people with psychosis rated the online intervention quick, feasible and easy to use, including finding materials they want, downloading or printing them, submitting answers and/or undertaking interactive exercises [105]. A web-based psychosis psychoeducational program for caregivers (iPEP) comprises web-based psychoeducational materials to facilitate self-learning of caregivers of patients with psychosis by also using an interactive online forum for sharing and dissemination amongst caregivers and communications with mental health professionals as well as face-to-face group-based psychoeducation to provide an offline interactive platform among the caregivers [7]. Overall, caregivers considered the iPEP website user-friendly and feasible [7]. The MOST software platform enlarged to integrate online therapy content for caregivers of patients with severe mental illness (SMI), particularly the application *Altitudes* was designed for early psychosis, including specific content designed to address the needs of caregivers of patients with FEP [53]. The MOST system has been considered safe for caregivers of young people with mental health problems by reducing family stress and correlations between those reductions and use of MOST system [53]. A protocol for an online RCT of a peer-supported online self-management intervention for education and providing a coping toolkit for relatives of people with psychosis or bipolar disorder has been published [54, 55] and further developed [56, 57, 58]. A parallel, two-arm feasibility RCT was designed to assess the knowledge and attitudes of relatives and caregivers regarding schizophrenia before, during, and after engaging with a non-commercial e-learning resource, focusing on African-Caribbean families with members affected by schizophrenia [59]. The intervention consisted of a web-based e-learning resource to enhance knowledge about schizophrenia and related psychoses amongst families and carers of African-Caribbean patients, called Culturally Appropriate Schizophrenia Psychological Education Resource (CaS-PER) [59]. An online RCT evaluated the effectiveness of an e-health intervention named Carers fOr People with Psychosis e-support (COPe-support) in promoting caregivers’ health outcomes, information and psychosocial support for caregivers of subjects affected with psychosis through the Internet, promoting flexible access and individualized choice [60]. Findings from the RCT indicated that the COPe-support intervention had a positive impact on the mental well-being and a variety of caregiving-related and mental health outcomes among participants; however, COPe-support intervention did not demonstrate superiority over the control treatment. At the same time, adherence was higher in participants who used the COPe-support system [61]. In the interview study with carers supporting individuals with psychosis using the COPe-support system, all participants reported a positive experience with COPe-support and promoted its wider implementation as a helpful adjunctive support resource for caregivers in the future [62]. A mixed-method, theory-driven implementation study employing a multiple case study design was conducted to develop and evaluate the Relatives Education and Coping Toolkit (REACT) program for individuals with psychosis or bipolar disorder. The study involved 281 staff accounts and 159 relatives’ accounts, with follow-up extending to 12 weeks [58]. Staff and family members expressed overall positive comments regarding the REACT program without any evidence that REACT would decrease staff time supporting relatives [58]. A single-blind RCT evaluated REACT alongside a resource directory against the combination of treatment as usual with the resource directory and the treatment as usual only, measured user distress and other wellbeing measures at baseline and at 12 and 24 months of those relatives of people with SMI across the UK who experienced higher levels of distress [56]. The findings indicated that an online self-management support toolkit, which includes a moderated group forum, is well-received by relatives and may provide a cost-effective, safe method of delivering support, encouraging their engagement as peers in care delivery [56]. Another pilot study assessed the feasibility and effectiveness of a Norwegian version of the web-based REACT (REACT-NOR) combined with phone-based support from trained family therapists. The study focused on evaluating how the service was perceived by relatives and family therapists, examining its impact on the relatives’ distress levels and expressed emotions, and identifying key facilitators and potential obstacles to integrating REACT-NOR into standard clinical practice [64]. The program was available 24 hours a day/7 day a week as a regular webpage. Differently from the original, REACT-NOR did not allow online interaction with the family therapists. A significant reduction in the level of expressed emotions from baseline to post-intervention (*p* = 0.03) with a simultaneous reduction of perceived stress level (*p* = 0.02), demonstrating that relatives experienced REACT-NOR as a tool they could use to adjust their own behaviour for both the patients and their own needs and an increased possibility to receive family interventions which may be limited due to a lack of consent from the patient, geographical distance or lack of resources [64]. A 3-phase study protocol was developed to specifically evaluate a culturally informed FAmily Motivational Engagement Strategy (FAMES) and implementation toolkit for coordinated specialty care providers to be applied to caregivers and family members of patients with FEP, with iterative mixed methods that will integrate qualitative and quantitative data and provide data on feasibility, acceptability and implementation outcomes [63]. A web-based psychoeducation course was developed to provide information and peer support for caregivers of individuals with psychotic disorders [65]. The course lasted 8 weeks and included 6 modules, each focused on a different theme: orientation, daily life, mental illness, patient and caregiver rights, treatment and wellbeing. Each module was one week long, except Orientation, which lasted 2 weeks [65]. Results confirmed the usefulness and the feasibility of this web-based psychoeducation course, particularly for those caregivers who have little experience as a caregiver. EOLAS programme is a recovery-oriented psychoeducation programme for psychosis designed for patients and their families [66]. During the COVID-19 pandemic, this programme has been carried out through online video conferencing platforms [66]. The structured programs consist of eight weekly group sessions, each lasting 90 minutes. These sessions cover topics such as psychosis, biopsychosocial treatment options, accessing services and support, as well as addressing stigma and promoting self-advocacy [66]. At the end of the study, findings suggested that the online version of this program is feasible, acceptable and useful for participants [66].

In Germany, recently, the first German-moderated online psychoeducation and support programme (ePSP) has been developed for family members of individuals experiencing early stages of psychosis [67]. This intervention has been set up as an online self-learning course on the platform Moodle, with evidence-based content about psychological interventions, peer-support principles in the context of family interventions for psychosis, self-care interventions, and communication and problem-solving skills learning programmes [67]. It comprises 5 modules: “What is Psychosis?”, “Treatment & Crisis”, “Communication & Emotion”, “Self-Compassion”, “Health Services” [67]. The information is offered through audio-visual material, texts and graphics. Throughout the intervention period, moderators could engage with participants in the module’s designated chats. After 2 weeks of participation, moderators sent each participants a private “check-in/motivation” email. Once the intervention period concluded, researchers reached out to participants to arrange their online post-intervention assessment appointment. Results have shown significant positive effects on perceived stress and beliefs about the illness [67].

### Studies on Virtual Reality-Assisted Interventions

A comparative clinical trial aimed at evaluating the benefits of combining CBT for voices followed by virtual-reality-assisted therapy (VRT) was carried out on ten patients with treatment-resistant schizophrenia or schizoaffective disorder, extracted from a larger comparative clinical trial comparing VRT vs CBT for voices. The trial, besides the small sample size, demonstrated significant improvements throughout time points on auditory verbal hallucinations, beliefs about voices, depressive symptomatology and symptoms of schizophrenia and quality of life following the sequence of combining both CBT and VRT compared to either intervention alone [68].

### Studies on Social Network Sites (SNS)-Mediated and Digitally-Based Psychoeducation Interventions Targeted to the General Population

A study examined three distinct psychoeducational interventions (focusing on medication, CBT, and psychodynamic psychotherapy) to assess their effectiveness in reducing stigmatizing attitudes toward individuals with schizophrenia in the general population. The findings described improvements in perceptions related to the dangerous unpredictability and anxiety associated with people living with schizophrenia [34]. A study investigated the comparative effectiveness of three 48-minute educational videos on YouTube in delivering psychosis-related psychoeducation to Chinese-speaking audiences [35]. Psychoeducational content included mental health-related topics about schizophrenia, early psychosis and FEP, and different psychosis treatment options. The study showed that the FEP video attracted the most viewer interest, achieving the highest view count, the greatest total watch time, the longest average viewing duration, and the highest number of shares [35]. A further study conducted by the same authors evaluated the efficacy of an Instagram advertisement promoting a YouTube video about FEP among Chinese-speaking individuals aged 18–34 by reporting an increasingly appealing attitude in viewing psychoeducation in the Chinese language focused on the signs and symptoms of FEP [36].

### Studies on Web-Based Psychoeducation Interventions Targeted to Patients with Psychosis or Schizophrenia

Psychoeducation is one of the most widespread and feasible psychosocial interventions for schizophrenia and relevant scientific evidence tends to show that, even when delivered via web, these programs are successful. For example, a quasi-experimental, feasibility and efficacy study evaluated a text-messaging intervention designed to identify early warning signs of relapse in patients with schizophrenia and named Information Technology Aided Relapse Prevention Programme in Schizophrenia (“ITAREPS”), reported a statistically significant reduction in the number of hospitalizations amongst those patients who were monitored with the prodromal signs M-Health service platform, compared to the period before the ITAREPS entry (*p* < 0.004). The ITAREPS demonstrated to be able to promote appropriate measures for early pharmacological interventions [69]. A quasi-experimental feasibility study compared patients and relatives who received an online multifamily group educational program with a cohort of patients and relatives who previously received treatment as usual [70]. The ‘Online Relative Support Group’ included a discussion board for participants and staff, links to relevant organizations and websites, a written and video psychoeducation intervention on behavioural family therapy, and a real-time chat-based intervention together with two optional real time chats/groups focused on medication and treatment issues and social support [70]. A further randomized controlled study compared the effectiveness of a web-based psychoeducation in a sample of patients affected with chronic schizophrenia hospitalized more than once to control group [71]. Steinwachs *et al*. [72] evaluated a web-based tool to help patients with schizophrenia in communicating with healthcare workers about evidence-based interventions compared to a control group. The intervention group was assigned to an interactive website with information on six domains (i.e., referrals, quality of life, medications, side effects, employment and family support), while the control group was assigned to a 22-minute video about schizophrenia treatment and a brochure including five treatment recommendations included by the website. Patients in the intervention group asked more questions about their medication and psychological and lifestyle issues to provide more information to clinicians. Moreover, they were more likely to check that they understood what the clinicians said by repeating or rephrasing the information for confirmation, and they were overly more dominant and respectful in their communication [72]. A cohort study collected qualitative and quantitative data from 93 patients’ evaluation reports in two psychiatric hospitals in Finland, following a web-based psychoeducation intervention consisting of six psychoeducation sessions, which were used over a period lasting between 1 and 70 days and took 10–360 minutes per patient. Psychoeducation sessions dealt with mental illness, treatment, wellbeing, patients’ rights, and daily life, and patients demonstrated, overall, a greater interest and positive attitude towards the web-based intervention [73]. A pilot trial of a mobile phone-based text message intervention called Mobile Assessment and Treatment for Schizophrenia (MATS), which provided health promoting behaviours by using CBT techniques, showed that interactive text message assessments and interventions are feasible and effective at enhancing medication adherence, socialization and auditory hallucinations in schizophrenia patients [74]. Outpatients with schizophrenia or schizoaffective disorder were randomized to intervention treatment (n = 75) consisting of the ITAREPS program or control group (n = 71) demonstrating a nine-fold reduction in the risk of hospitalization in the intervention group compared to the control group [75]. A web-based support system which allows routine outcome monitoring more accessible to patients with schizophrenia, by showing a valuable potential of the tool in improving routine outcome monitoring practice for psychotic patients [76]. An uncontrolled single-group study aimed at evaluating feasibility, safety, acceptability and initial benefits of a pilot web-based psychosocial intervention named “Horyzons”, which adopts the ‘MOST’ conceptual model by comprising a platform for delivering evidence-based and interactive psychosocial interventions which are increased by a moderated online social networking environment [77]. Findings showed that participants provided positive ratings for the ease of use, enjoyment and perceived utility [77]. A pilot study evaluated a web-based CBT for auditory hallucinations in subjects with psychosis by reporting the feasibility and efficacy of the intervention in coping with voices, reducing the severity of voices and other psychotic symptoms, and overall psychopathology [78]. A pilot study developed a website, used by mental health workers, to improve therapeutic sessions about personal recovery along an 8-session low intensity intervention addressed to individuals with persisting psychotic disorders by demonstrating an improvement in personal recovery by an average standardized effect (d = 0.46) and overall feasibility and acceptability by users [79]. An RCT of a digitally assisted low intensity intervention to promote personal recovery in persisting psychosis named Self-MAnagement and Recovery Technology (SMART) was developed in a research protocol [80]. A pilot study of a neuroplasticity-based online social cognitive training program (named “SocialVille”) addressed to young people with schizophrenia demonstrated feasibility and resulted in improvements in social functioning and motivation [81]. A pilot study assessed the applicability and efficacy of a novel online training program on social cognition for schizophrenia patients named “e-Motional Training”, by reporting a statistically significant efficacy after the intervention at the Ekman 60 Faces Test, at the Theory of Mind tests and Ambiguous Intentions Hostility Questionnaire [82]. A protocol of an RCT of a moderated online social therapy (Horyzons) developed to improve social functioning and maintain clinical gains from specialist FEP services was proposed by Alvarez-Jimenez *et al*. [83]. A moderated online intervention to treat social anxiety in FEP individuals named EMBRACE demonstrated to be feasible, acceptable and a safe online intervention targeting specifically social anxiety as a primary treatment concern in youth with FEP [84, 85]. A pilot study of a home-delivered web-based intervention of cognitive remediation targeted to FEP individuals reported an overall positive perception among participants, a significant improvement in sustained attention, verbal memory, negative and positive symptomatology and the overall functioning after 6 months [86]. An evaluation of an adapted version of the Horyzons program, initially designed in Canada to facilitate relapse prevention and recovery in young adults experiencing FEP, revealed participant concerns regarding the program’s practical implementation. These concerns primarily focused on resource availability (e.g., internet speed, site moderation, crisis management) and the need to enhance the platform’s usability and accessibility through mobile devices [87]. The pilot study of Horyzons-Canada has demonstrated that this intervention is feasible, acceptable, safe and potentially effective [87, 88, 89]. At the same time, it could help maintain patient functioning and prevent worsening [88, 89]. An RCT administered an online intervention for psychosis by reporting worrying and sleep problems as predictor variables of psychotic symptom variability during the online intervention [90]. A secondary analysis on voice hearers from the EVIBaS program evaluating a psychological online intervention (POI) for psychosis, by indicating that the POI is likely to improve mindfulness among psychotic participants [91]. A moderated online social intervention for FEP individuals integrating the moderated Horyzons platform for 12 weeks demonstrated a significant improvement in psychotic symptomatology and loneliness levels [92]. A double-blind, controlled, multi-site RCT administered an online social cognition training in schizophrenia patients (SocialVille) and control group by reporting significant improvement in social functioning on the virtual functional capacity and motivation subscale in the schizophrenia group assigned to intervention, compared to the control group [93]. A web-based digital resource designed for use on tablet computers was created for peer work sessions. It included 14 videos grouped into six overarching themes (i.e., My Journey, Self-Care, Connections, My Identity, Life, and Mental Health for FEP) [94]. A pragmatic protocol of a RCT, single blind web-based intervention using short message service (SMS) was designed to improve transitions from the emergency department to evidence-based early psychosis intervention services [95]. A randomized, single blind, controlled study of a standardized computer-assisted cognitive remediation (CACR) program administered to young people affected with psychosis or schizophrenia by comparing outcomes following the intervention program and those coming from treatment as usual [96]. Findings evaluated the differences in terms of improvement in cognitive functioning (i.e., visual learning, speed of processing, reasoning and problem solving, attention/vigilance, verbal learning, and working memory), mental wellbeing and symptomatology improvement of patients; perceived competence in occupational functioning; and engagement in occupational roles like worker, student, trainee, or homemaking. Overall, the CACR program is generally successful in enhancing participants’ cognitive abilities, particularly in areas like verbal learning, processing speed, and attention or vigilance. Significant improvements in mental wellbeing and significant positive changes in the functional states of young participants have been observed in the intervention group, compared to the control group [96].

### Effectiveness

The comparative effectiveness research (CER) tool has been used to assess the quality of the studies included in this systematic review. We identified 14 studies that evaluated specifically the effectiveness of digital interventions for schizophrenic psychosis. However, after study design and cohort selection quality were assessed, 12 out of 14 studies showed high quality indicators regarding criterion 1 (i.e., right patients enrolled), 12 studies regarding criterion 2 (i.e., right treatments administered), 11 studies regarding criterion 3 (i.e., right outcome(s) investigated) and 12 studies regarding criterion 4 (i.e., right timing considered) (see also Table 4, Ref. [3, 53, 55, 56, 59, 60, 61, 64, 70, 74, 80, 82, 94, 95]). While regarding the appropriateness of the data source, 11 out of 14 studies showed high quality indicators regarding criterion 1 (i.e., data source meets study aims), 11 studies regarding criterion 2 (i.e., significant representative sample). Finally, when the rigour of the study methodology was assessed, 11 out of 14 studies were evaluated of high-quality concerning criterion 1 (i.e., new initiators of the treatment are targeted by study methodology), criterion 2 (i.e., presence of comparator(s)), criterion 3 (i.e., bias control), while 10 studies regarding criterion 4 (i.e., sensitivity analyses performed).

**Table 4.** **Comparative effectiveness research (CER) tool – quality indicators**.

Study	1. Study design and cohort selection				2. Appropriateness of the data source		3. Rigor of the study methods			
	1.1.	1.2.	1.3.	1.4.	2.1.	2.2.	3.1.	3.2.	3.3.	3.4.
[3]	Y	G	G	G	G	G	G	G	G	Y
[53]	G	G	G	G	G	G	G	G	G	G
[55]	G	G	G	G	G	G	G	G	G	G
[56]	G	G	G	G	G	G	G	G	G	G
[59]	G	G	G	G	G	G	G	G	G	G
[60]	G	G	G	G	G	G	G	G	G	G
[61]	G	G	G	G	G	G	G	G	G	G
[64]	G	Y	Y	G	Y	R	Y	R	R	R
[70]	Y	Y	Y	Y	Y	Y	R	R	R	R
[74]	G	G	G	G	G	G	G	G	G	G
[80]	G	G	Y	Y	Y	R	Y	Y	Y	R
[82]	G	G	G	G	G	G	G	G	G	G
[94]	G	G	G	G	G	G	G	G	G	G
[95]	G	G	G	G	G	G	G	G	G	G

***Principles***. 1.1.=Are the right patients being studied?; 1.2.=Are the right treatments being studied?; 1.3.=Are the right outcomes being studied?; 1.4.=Is the right timing being used for the study?; 2.1.=Does the data source meet the needs of the study aims?; 2.2.=Does the study include a sufficient number of patients to ensure statistical power to address a clinically meaningful effect size?; 3.1.=Does the study methodology target new initiators of the treatment?; 3.2.=Are the comparator cohort(s) included in the study from the same time period as the main intervention?; 3.3.=Does the analysis include careful consideration and application of appropriate techniques to control for potential bias?; 3.4.=Are sensitivity analyses performed to assess robustness of the findings?. ***Quality indicators***: G, high degree; Y, moderate degree; R, low degree.

## Discussion

Overall, all studies included in the present review evaluated the effectiveness of psychosocial and/or psychoeducational interventions targeted to patients with schizophrenia and/or their family members. Studies here retrieved demonstrated an overall high-quality methodology, according to the CER tool. Psychosocial interventions, either family- or individual-based, are considered beneficial and recommended for schizophrenia as adjunctive treatment to psychopharmacology [10, 11, 106], with psychoeducation being among the most effective approaches for improving compliance and reducing the relapse rate in psychosis [107]. Psychoeducation can be delivered via individual or group-based interventions and could involve clinicians who can take the role of information provider [107]. Furthermore, multi-component psychosocial interventions comprising peer support, psychoeducation, education about coping strategies and problem-solving techniques for schizophrenia spectrum disorder management have been implemented and effectively provided [98].

In our review of current published literature on this topic, we confirmed good effective outcomes of digitally-delivered psychosocial interventions when addressed to patients and their caregivers/parents/family members [37, 66, 70, 104] only to individuals affected with schizophrenia [38, 40, 69, 71, 72, 81, 82, 88, 89, 93], or to family members or caregivers of patients with schizophrenia [54, 55, 56, 57, 58, 59, 61, 62, 64, 65, 67] and psychosis [56, 108]. We found that digitally-based approaches were associated with good clinical and functioning outcomes within the psychosis spectrum [39, 40, 41, 42, 43, 76, 90, 91, 97] and even in FEP patients [3, 7, 44, 45, 77, 83, 84, 85, 86, 89, 92, 98, 109]. Current published literature describes that some studies were developed and implemented to address both patients and their caregivers/parents/family members [52, 66, 69, 110], while other studies were performed only to be provided to individuals/patients affected with schizophrenia [37, 39, 68, 70, 71, 80, 81, 92], spectrum psychotic disorders [38, 40, 41, 42, 75, 87, 90, 107], or FEP patients [3, 43, 44, 76, 82, 83, 84, 85, 88, 89, 91, 97, 98]. While other digitally-based psychosocial interventions have been specifically designed for family members or caregivers of patients with schizophrenia [53, 54, 55, 56, 57, 58, 63], psychosis [55, 61, 62, 65, 67, 109] or FEP [7, 98]. Only few studies specifically testing digitally-delivered (mainly psychoeducational) interventions on the general population [34, 35, 36].

Notably, many digitally-based interventions were effectively provided following highly specific and structured programs [37, 42, 43, 53, 61, 62, 65, 66, 67, 69, 75, 77, 81, 83, 88, 89, 93, 98, 108, 111]. The Schizophrenia Guide software represents one of the first telehealth applications designed for schizophrenia individuals and their support persons in their homes and group-based multifamily therapy online [37]. The ITAREPS was designed to identify early warning signs of schizophrenia relapse [69, 75]. The Australian SMART program was developed to promote personal recovery and self-management of mental health in individuals with a history of psychosis by administering six modules [42, 43, 85]. Other studies proposed a MOST approach by integrating online therapy and moderated social networking functions [53, 77, 83, 98, 111]. The moderated social networking functions include a personal profile page, a network, group problem solving, discussion threads linked to the modules and a ‘job zone’ which provides vocational information [53, 77, 83, 98, 111]. The “Horyzons” program includes a peer-to-peer social networking, a tailored therapeutic intervention, an expert and peer moderation, and a new model of psychological therapy (strengths and mindfulness-based intervention) targeting social functioning to FEP patients [44, 45, 83, 87, 88, 89, 92]. The SocialVille aims to address deficits in social cognition by applying neuroplasticity-based learning principles, focusing specifically on the brain systems that support social cognition and may be impaired [81, 93]. The SocialVille exercises target stimulus representation and processing speed in the specific neural systems involved in social cognition [81, 93]. The e-Motional Training (ET®) program is an online, clinician-supervised social cognition training consisting of 12 one-hour sessions conducted weekly. It includes modules focused on emotional perception, an animated cartoon component for the theory of mind, and exercises in attributional style with automated metacognitive feedback [82]. Meanwhile, the EViBaS program is a CBT-based online self-help intervention designed for individuals with psychosis. It provides modules addressing delusions, voice hearing, social skills, and mindfulness, with participants receiving guidance throughout the program from a dedicated moderator [90, 91, 97]. The EVIBaS program’s POI includes a mindfulness module [90, 91, 97]. The EMBRACE moderated online social cognitive behavioural intervention which includes expert and peer-moderation together with a CBT-based treatment model for social anxiety in FEP individuals, relevant literature about psychosis and its clinical correlates (including social anxiety, paranoia, social rank and shame), feedback from the youth focus group, and a highly multidisciplinary collaborative approach to design therapy comics [84, 85]. The CACR program comprises a standardized cognitive remediation program (CACR), which includes two sessions of psychoeducational talks, sixteen sessions of a computerized cognitive training program, and four bridging sessions [96]. Whilst other studies were designed to apply a mobile app-based or virtual-reality-assisted psychosocial intervention [3, 6, 52, 68].

Furthermore, a set of studies were specifically designed to be offered to caregivers and/or family members of individuals with psychosis and/or schizophrenia [7, 53, 54, 55, 58, 59, 60, 61, 62, 64, 65, 67, 98, 99, 105, 108, 112, 113, 114, 115]. For instance, the e-Sibling Project is a comprehensive online intervention comprising 4 modules (information on psychosis, coping and promoting wellbeing strategies, siblings’ blogs and discussion forum with peers, and “ask the experts” function), designed to be addressed to siblings of individuals affected by psychosis [114]. The iPEP program was developed aiming to offer up-to-date online information about psychosis and available community resources, based on 19 articles covering detailed information on etiology of psychosis, different treatment modalities, recovery relapse, medication side effects, and risk management as well as skills for caregiving and self-care including communication skills with patients, skills handling common difficult situations (e.g., when the patient refuses treatment, when the patient has a poor insight and/or lack of motivation) and caring for themselves when feeling stressed [7]. Within the MOST software, the application Altitudes was specifically designed for caregivers of FEP patients and comprised 10 separate pathways, including content on psychoeducation, self-care, strategies to cope with stress related to caregiving, and communication with the youngsters [53, 98, 111]. The CaS-PER program is composed of 13 fact-based topics on psychosis (i.e., schizophrenia and black Caribbean people in the UK, family and relationships, symptoms, recovery and illness management) and 9 imaginary stories highlighting important aspects of factual information from the perspectives of key stakeholders (e.g., healthcare professionals, family members, service users, and the police) [59]. The COPe-support is an e-health intervention dedicated to family members of individuals with psychosis aimed at providing psychoeducation and emotional support using healthcare professional contribution and peer support [60, 61, 62]. The REACT toolkit consists of 12 evidence-based psychoeducation modules, peer support via a group forum, and a confidential messaging service for relatives of people with psychosis or bipolar disorder [56, 57, 64]. The FAMES program is a multi-site, mixed methods project composed of three phases: (a) intervention development; (b) intervention modification; (c) efficacy evaluation using a non-randomised stepped-wedge pilot trial design. FAMES involve early, continuous and motivational contact, which includes motivational techniques [61].

In addition, studies investigating the perceived patients’ experiences regarding digital psychosocial interventions reported a great reduction in individual stress levels, and high levels of perceived social support and participation [37, 42, 43, 52, 62, 65, 67, 73, 76, 81]. Digitally-delivered interventions were overly considered useful, feasible and helpful in coping with alienation and isolation derived from the illness [38, 39, 40, 44, 45]. A further study investigating patients’ knowledge and attitudes towards digital interventions, including CBTp, is still ongoing, without any published findings available [41, 97]. Overall, subjects with schizophrenia displayed a higher Internet usage and demonstrated easy use of text-based telehealth applications to receive psychosocial treatments, and psychoeducation and to participate in group and multi-family therapy [37], with substantially increased engagement amongst those patients receiving emails as supportive and motivating tools [42, 74, 95]. Parents and caregivers who received a digitally-based psychoeducation intervention overly reported increased perceived usefulness and feasibility [7, 56, 57, 62, 64, 65, 67, 105], even though a study did not report substantial differences when compared to in-person delivered family support [37]. However, in this field, other ongoing studies are still in the recruitment and analysis stage, as they were published only as research protocols and, hence, could potentially help in drafting more definitive conclusions [59, 63].

Currently, internet- or mobile-based treatments have also garnered increasing interest and usage by augmenting traditional face-to-face interventions [23]. In fact, the Internet and online devices (including smartphones and digital tools, such as VR) may represent potentially transformative tools, which may deliver a wide range of mental health services, including digitally-based/digital adjunctive psychosocial interventions, psychoeducation, self-management and support for both patients with psychosis and schizophrenia and their caregivers [42, 43]. Both web- and mobile-based psychosocial and psychoeducational interventions appear feasible, acceptable and potentially effective in improving clinical outcomes for individuals with schizophrenia and psychosis [3, 116]. Moreover, e-mental health could be particularly useful in overcoming the gap between mental health services and subpopulations such as youngsters, as reported in a study in which it has been reported that 85% of young adults diagnosed with FEP agreed or strongly agreed to use YouTube and other social network sites for mental health education, counselling and support [15] and in a recent survey carried out on psychotic patients [17]. Furthermore, people who tend to avoid treatment due to cultural stigma could benefit from psychoeducation and psychosocial interventions via the Internet and social network sites [35, 36, 52].

Despite the limitations of preliminary research, data suggest that digitally-delivered mental health interventions for schizophrenia and psychosis are generally feasible and acceptable, particularly for symptom monitoring and clinical management, as well as for improving sociability and treatment adherence [6, 12, 21, 37, 73, 76, 116, 117, 118, 119, 120]. However, it has also been argued that digital interventions, when provided not as adjunct interventions to treatment as usual/in-person care and treatment, particularly when proposed to FEP individuals, could not be fully effective in promoting treatment adherence [2]. Therefore, a preliminary assessment to predict the level of user engagement, involvement and interaction with digital interventions should be preliminarily and carefully performed before proposing a digitally-based psychosocial and/or psychoeducational intervention to a patient with psychosis or schizophrenia, as already recommended by previous studies [14, 121]. In fact, the engagement and interaction level may be influenced by individual, environmental, clinical and intervention factors, including low digital literacy, limited Internet access and technical equipment which may be secondary to a low socio-economic status; the level of cognitive impairment and positive and negative symptomatology (i.e., whether a patient is in an acute and/or subacute phase of illness) [14, 74, 112, 122, 123]. Moreover, digitally-delivered psychosocial and psychoeducational activities, particularly for those patients with psychosis and/or schizophrenia, may require adequate levels of safety, protection and oversight, as these individuals may own personality traits that jeopardize safety within a digital platform and display suspiciousness, which may be exacerbated in an online environment [6]. Therefore, clinicians should preliminarily investigate the type of patient’s ‘digital engagement’, i.e., ‘active’ (e.g., requiring more direct user participation such as involvement in self-monitoring assessments, text-message to his/her clinician, etc.), ‘passive’ (e.g., requiring less direct user’s participation such as watching a video, reading an exercise, etc.) or ‘blended’ (e.g., a mixed virtual hybrid psychiatrist-patient relationship), before deciding to propose a digitally-delivered psychosocial and/or psychoeducation interventions [121, 124].

In addition, clinicians should consider the impact of a digitally-delivered intervention on medication and treatment adherence, particularly when they decide to propose an online psychosocial intervention to patients with psychosis and/or schizophrenia, particularly with limited family support or involvement and/or without possible remote support and assistance [14, 53, 97, 112]. With this regard, a retrospective chart review study investigated the role of family support and telehealth delivery in predicting medication and treatment adherence in Youth with FEP by demonstrating a more likely chance to disengage from medication amongst those patients who were treated with telehealth compared to people who received face-to-face mental health care (*p* = 0.0177) [2]. Providing an adjunct email, SMS reminders or telephone support may encourage and more likely to engage patients with psychosis and schizophrenia in self-guided online psychosocial and/or psychoeducational interventions [17, 37, 42, 52, 57, 58, 69, 74, 125, 126, 127]. Overall, the maintenance of engagement and adherence to digital interventions over time may require a process comprising an internalization of values and skills required for change, self-determination, a more integrated recovery style and higher levels of motivation and adherence to assignments, compared to traditional face-to-face interventions [14, 42, 43].

Digital interventions for families may be an alternative or combined with in-person ones. Family caregivers and parents may often neglect physical and psychosocial needs of individuals affected with psychosis and schizophrenia; they may be concerned about the course and prognosis of their relative’s illness and potential for recovery as well as they may experience high levels of anxiety, tension, stress and uncertainties and, hence, they may not adequately be helpful in managing illness by family members and/or caregivers of individuals affected with schizophrenia and psychosis [9]. Therefore, family interventions (i.e., problem solving skills training, psychoeducation, cognitive appraisal and stress management) may help in overcoming stressful interpersonal environment within the family context that may indirectly exacerbate psychotic symptoms, facilitate premature and recurrent relapses, hospitalizations and influence treatment adherence of the patients affected with psychosis or schizophrenia [67, 107]. Studies focusing on web-based family psychosocial interventions reported significant reductions in hospitalizations, early warning signs identification, higher satisfaction levels and decreased distress levels amongst patients’ relatives [64, 69, 70]. However, most of the studies here retrieved specifically designed to administer family-based interventions to relatives and caregivers of individuals with psychosis and schizophrenia are still in the preliminary phase, being pilot research protocol studies without published findings [59, 95, 113]. At the same time, there may be challenges in accepting such interventions, especially in contexts where there is no digital education, both for families and doctors. The cost of digital devices might also not be so affordable for families of these patients, increasing the gap towards poorer families and denying them access to potentially effective interventions.

Although the evidence of the potential benefits of digitally-delivered psychosocial interventions, the included studies have some limitations. Some studies had small sample sizes or were conducted in specific populations, limiting the generalization of results to the general population. Furthermore, some studies used a wide range of digital tools, intervention durations, and therapeutic approaches, as well as short follow-up periods. This heterogeneity makes it challenging to define consistent comparisons or establish clear conclusions about the relative efficacy of specific interventions, relapse prevention or sustained improvements in quality of life. At the same time, some studies lack direct comparison with face-to-face approaches or published results, making it unclear how effective the intervention is. Finally, some studies had methodological weaknesses (such as lack of randomization, small sample sizes, or insufficient control groups) that may lead to methodological bias, limiting the reliability of the study and hiding the significant effect of the intervention studied.

## Conclusions

In conclusion, findings so far published seem to indicate an undoubted efficacy and effectiveness of digitally-delivered psychosocial and/or psychoeducational interventions both to patients and their family members, as an adjunctive strategy in those cases with a good digital literacy level, with Internet access and facilities, with a cooperative family support. Further studies are needed to clearly compare the effectiveness of in-person and digitally-delivered interventions and to better deepen and identify which main determinants may influence the level of attractiveness, engagement, and treatment adherence to digital modality in schizophrenia and psychotic patients. Furthermore, it would be useful to conduct studies comparing different digital interventions with the same objectives, e.g. web-based and app-based interventions. Moreover, one could argue that there is also the need to better understand and define which are the patient’s basic (cognitive, technical and emotional) skills needed to accept digitally-delivered interventions effectively and whether there are some differences in treatment adherence according to the type of interventions, target populations and different illness phase. Knowing these patient characteristics would allow us to better target the type of intervention, increasing the response to treatment. Interventions that are too elaborate may not be effective for patients with major cognitive problems, so simpler interventions should be preferred. Therefore, further comparative randomized controlled trials (RCTs) but also real-world setting studies should be carried out in order to digitally characterize the ‘ideal’ patient and the ‘ideal’ family who could benefit from digital psychosocial and/or psychoeducational interventions in the field of schizophrenia and psychotic area. Finally, further studies should be carried out on digitally-delivered psychoeducational interventions specifically targeted to the general population in order to increase illness awareness and provide informative anti-stigma tools on schizophrenia and psychosis.

## Availability of Data and Materials

All data generated or analyzed during this study are included in this published article.

## Supplementary Material

Supplementary material associated with this article can be found, in the online version, at https://doi.org/10.62641/aep.v53i2.1851.
